# The crosstalk of monocyte-neutrophil in hair follicles regulates neutrophil transepidermal migration in contact dermatitis

**DOI:** 10.1038/s42003-025-07960-w

**Published:** 2025-04-04

**Authors:** Zhan Fan, Yilun Xu, Yafang Lu, Xinlin Li, Mengli Xu, Jinxin Liu, Zhenzhen Cai, Jiayang Liu, Jingping Su, Jialu Wang, Qingming Luo, Zhihong Zhang, Zheng Liu

**Affiliations:** 1 School of Life and Health Sciences, Hainan Province Key Laboratory of One Health, Collaborative Innovation Center of One Health, Hainan University, Haikou, Hainan China; 2 State key laboratory of digital medical engineering, School of Biomedical Engineering, Hainan University, Sanya, Hainan China; 3 Britton Chance Center and MOE Key Laboratory for Biomedical Photonics, Wuhan National Laboratory for Optoelectronics-Huazhong University of Science and Technology, Wuhan, Hubei China

**Keywords:** Inflammation, Imaging the immune system, Neutrophils

## Abstract

The excessive accumulation of neutrophils within the epidermis is a significant hallmark of cutaneous diseases; however, the mechanisms governing neutrophil transepidermal migration (NTEM) remain inadequately understood. In this study, we develop trichromatic-fluorescence-labeled chimeric mice by utilizing *Cx3cr1*^GFP/+^*Lyz2*^RFP/+^ mice as bone marrow donors and Krt14^YFP/+^ mice as recipients. This approach enables us to visualize the process of NTEM and the crosstalk between neutrophils and monocytes in a murine model of irritant contact dermatitis (ICD). Intravital imaging reveals a preferential transmigration of neutrophils through hair follicle (HF), where dermal neutrophils exhibit limited mobility and interact with dermal monocytes. Notably, 18 h following hapten exposure, dermal neutrophils continuously migrate toward HF regions and form clusters within 3 h. Importantly, MMP-9 is identified as essential for the NTEM process; the depletion of dermal monocytes results in a significant reduction of MMP-9 expression in the skin and inhibits the NTEM process in ICD. Mechanistically, dermal monocytes are found to be a crucial source of the cytokines TNF-α and CXCL2, which promote the upregulation of MMP-9 in neutrophils. Therefore, our results highlight HF regions as crucial gateways for dermal monocyte-modulated NTEM and provide visual insights into the crosstalk between neutrophils and monocytes in inflammatory skin disorders.

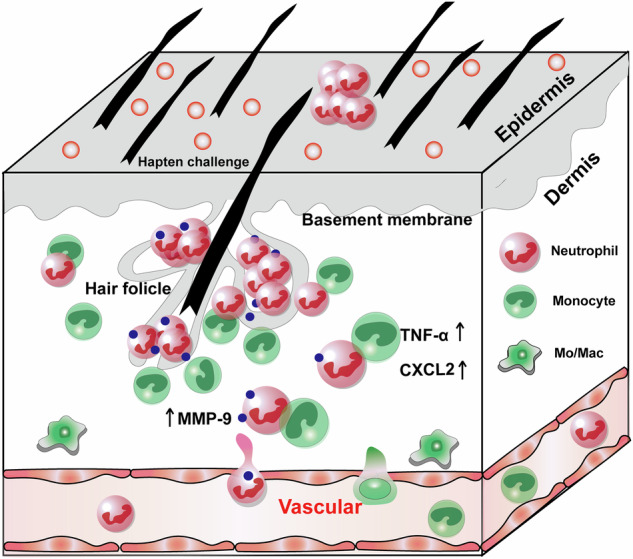

## Abstract (web summary)

Intravital imaging reveals that dermal monocytes orchestrate neutrophil transepidermal migration through hair follicles in skin inflammation via TNF-α and CXCL2, driving enhanced MMP-9 expression in neutrophils for epidermal infiltration.

## Introduction

Neutrophils are the most abundant immune cells in human blood and are indispensable components of immune defense^1^. They act as double-edged swords by playing essential roles in clearing infection and wound healing as well as causing tissue damage^2^. Neutrophil deficiencies result in chronic or cyclic neutropenia and chronic granulomatous disease (CGD), indicating the indisputable essential role of neutrophils in immunity^3^. Conversely, dysregulation of neutrophils contributes to inflammatory diseases, severe allergies, and autoimmunity^4–6^.

Neutrophil transepithelial migration and accumulation at mucosal surfaces is a hallmark of many inflammatory conditions including cutaneous disease^7^. Skin serves as a direct barrier between the human body and the external environment which is constantly stimulated by a variety of exogenous microorganisms, small molecular compounds, and so on^8,9^. The infiltration of neutrophils into the epithelium is one of the key steps in mediating cutaneous, respiratory, and bowel disease^10,11^. Neutrophils infiltrated in the epidermis may exacerbate inflammatory skin disorders such as psoriasis, atopic dermatitis, and contact dermatitis (CD)^12–14^. Contact dermatitis(CD) is one of the most common inflammatory dermatological conditions, with irritant contact dermatitis (ICD) accounting for 80% of cases^15^. ICD is caused by direct cellular toxicity leading to the inflammation and activation of the innate immune system. Irritants may also excite nociceptors which cause the release of vasoactive peptides such as substance P and subsequently induces vasodilation and the infiltration of diverse leukocytes including neutrophils and inflammatory monocytes from the blood into the skin^16,17^. However, the mechanism by which neutrophils transmigrated from dermis to epidermis remains unclear.

Finely controlled neutrophil infiltration in the epidermis is essential for the homeostasis of epidermal immunity. Therefore, the study to visualize neutrophil migration across the epidermis and its regulatory mechanism are of great value for analyzing the pathogenesis and exploring alternative therapies for skin immune diseases.

After the pathogen challenge, skin-resident immune cells release inflammatory factors and chemokines, inducing the extravasation of neutrophils and monocytes into the dermis^18^. The infiltration of neutrophils at the dermal-epidermal junction further leads to epidermal injury and triggers a strong inflammatory reaction^19^. Several studies uncovered the intercellular communication and cooperation among neutrophils and other cells which facilitate neutrophil transepithelial migration. In the intestine, previous studies on the neutrophil transepithelial migration found that the neutrophils migrating to the tight junctions first encountered the intestinal epithelial cells (IEC) basolateral surface in the basal layer of the epithelium^20^. The encounter of neutrophils and intestinal epithelial cells (IEC) mediates intracellular signal transduction and phosphorylation of connexin in epithelial cells and increases the permeability of the epithelial region^21,22^. In our previous study, we have visualized T cell-regulated monocyte clusters around the HF region which mediate keratinocyte death in acquired cutaneous immunity^23^. We wondered whether monocytes around HF regions also play a key role in tuning the transepidermal migration (TEM) of neutrophils in innate cutaneous immunity.

Intravital imaging of leukocyte behavior and function under inflammatory microenvironments has greatly advanced the understanding of the spatiotemporal dynamic information of cellular cooperation and molecular events involved in neutrophil migration. It provides valuable intuitive evidence for analyzing the mechanism of neutrophil-mediated inflammatory diseases. For instance, Tim Lämmermann et al. found that colony swarm behavior migrated to the damaged area of neutrophils in the aseptic injury of the dermis^24^. Sreeramkumar et al. found neutrophils polarization and scan for activated platelets during extravasation from vessels to initiate inflammation^25^. Although the molecular events and dynamic processes regulating neutrophil extravasation from blood vessels and the interstitial migration of neutrophils have been well studied, there is still a lack of visualized evidence in vivo regarding the process of NTEM in inflamed skin. Quantitatively characterizing and extracting the motion parameters and morphological characteristics of neutrophils and monocytes may uncover the details of NTEM and subsequent neutrophil-mediated cutaneous diseases.

Here, we established trichromatic-fluorescence-tagged BM chimeric mice to visualize the dynamic interplay of neutrophils, monocytes, and keratinocytes in a murine ICD model. Our results explored the dynamic process of NTEM and revealed that dermal monocytes regulate neutrophil infiltration in the inflamed epidermis. This in situ visualization offered intuitive evidence for our understanding of the monocyte-mediated NTEM in cutaneous immunity.

## Results

### Simultaneous visualization of monocytes, neutrophils, and keratinocytes by reconstitution bone marrow chimeric reporter mouse

**Fig. 1:**
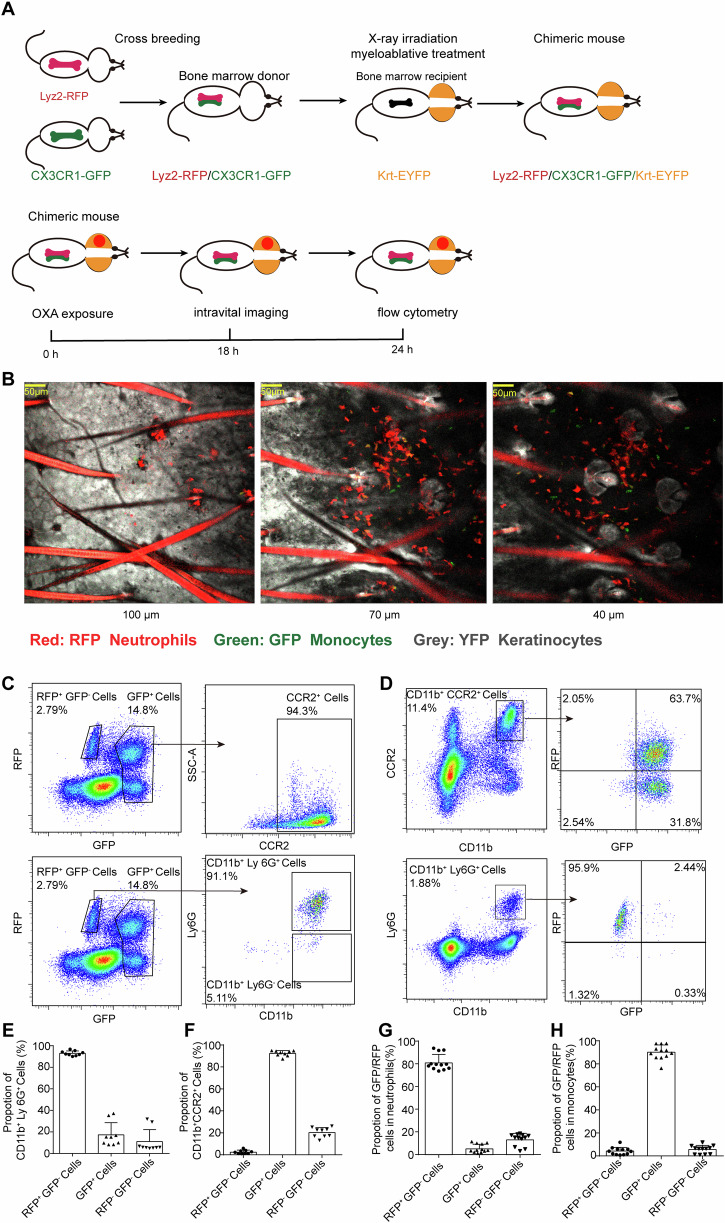
Reconstructing endogenous trichromatic-fluorescence-labeled chimeric mice. **A** Schematic representation of the Krt14-YFP/Lyz2-RFP/CX3CR1-GFP BM chimeric mouse reconstitution and the OXA-induced ICD procedure. **B** Representative confocal images of OXA-challenged chimeric mouse skin in different depths. GFP (monocytes), RFP (neutrophils), EYFP (keratinocytes), scale bar: 50  μm. **C**, **D** representative flow cytometry images of skin-infiltrated myeloid cells. **C** The proportions of monocytes/neutrophils in GFP^+^ or RFP^+^ cells. **D** Proportions of GFP and RFP expression in monocytes and neutrophils. **E** Quantitative of neutrophils proportions in RFP^+^GFP^-^ cells. **F** Monocytes proportions in GFP^+^ cells. Quantitative data of RFP^+^ or GFP^+^ proportions in neutrophils (**G**) and monocytes (**H**). *n* = 9 in (**E**, **F**), *n* = 12 in (**G**, **H**). All the mice were female for these experiments. Data are presented as mean ± SD from three independent experiments.

It is previously assumed that monocytes/macrophages may exert control over neutrophils’ transvascular or transepithelial migration in certain pathology conditions^18^, but a coherent model to study the role of monocytes in mediating NTEM during cutaneous inflammations is presently missing. Here, *Lyz2-*cre mice and Ai14 mice were crossed to obtain *Lyz2*-RFP heterozygote mice, while Krt14-cre mice and R26R-EYFP were crossed to obtain Krt14-EYFP heterozygote mice. Next, *Cx3cr1*-GFP, *Lyz2*-RFP, and Krt14-EYFP mice were utilized to construct the trichromatic-fluorescence-tagged bone marrow (BM) chimeric mouse. As seen in Fig. 1A, we crossed the *Lyz2*-RFP mouse with *Cx3cr1*-GFP mouse to obtain *Lyz2*^RFP/+^*Cx3cr1*^GFP/+^ mice as bone marrow donors and X-ray irradiated Krt14-EYFP mice were used as recipients to build the trichromatic-fluorescence-tagged chimeric mouse.

Next, we tested whether the trichromatic-fluorescence-tagged chimeric mouse efficiently and accurately distinguished keratinocytes, neutrophils, and monocytes in inflammatory skin in vivo. Here, an oxazolone (OXA)-induced ICD model was used to investigate innate immune cell behavior^26,27^. Our data showed that recruited cells in OXA-challenged skin were mostly monocytes and neutrophils 24 h post OXA exposure (Supplementary Fig. 1A, B). The ear thickness was elevated 18–48 h post OXA exposure, and the average ear thickness in OXA-treated mice was 302.22 μm at 18 h and 353.33 μm at 24 h post OXA challenge (Supplementary Fig. 1A). In the OXA-treated group, the total number of neutrophils and monocytes infiltrating the skin (including both epidermis and dermis) was 94,640 ± 20,930 and 43,180 ± 8320, respectively. In contrast, neutrophils and monocytes were rarely observed in the vehicle (AOO)-treated group (Supplementary Fig. 1B). In the trichromatic-fluorescence-tagged chimeric mice, skin was infiltrated with BM-derived neutrophils and monocytes, which can be distinguished by EGFP and RFP two weeks post-irradiation reconstitution, and the keratinocytes in the epidermis were labeled with EYFP (Fig. 1B). Flow cytometric data further confirmed the labeling accuracy of monocytes and neutrophils in the skin (Fig. 1C, D). As seen in Fig. 1E, over 93.02% RFP^+^GFP^−^ expressing cells were identified as CD11b^+^Ly6G^+^ neutrophils; while in GFP^+^ cells (regardless of RFP expression), 92.42% of which were identified as CD11b^+^CCR2^+^ monocytes in the ICD model (Fig. 1E, F). Vice versa, CD11b^+^Ly6G^+^ neutrophils were mostly RFP^+^GFP^−^ (81.18%) expressing cells (Fig. 1G), and CD11b^+^CCR2^+^ monocytes were mostly GFP (90.13%) expressing cells (Fig. 1H). Thus, the chimeric mouse ensured the labeling accuracy of dermal monocytes (GFP^+^), neutrophils (RFP^+^GFP^−^), and keratinocytes (YFP^+^) in the mouse ICD model, which proved to be a competent tool to study monocyte-neutrophil behavior as well as NTEM in vivo.

### Depletion of monocyte alleviating NTEM

**Fig. 2:**
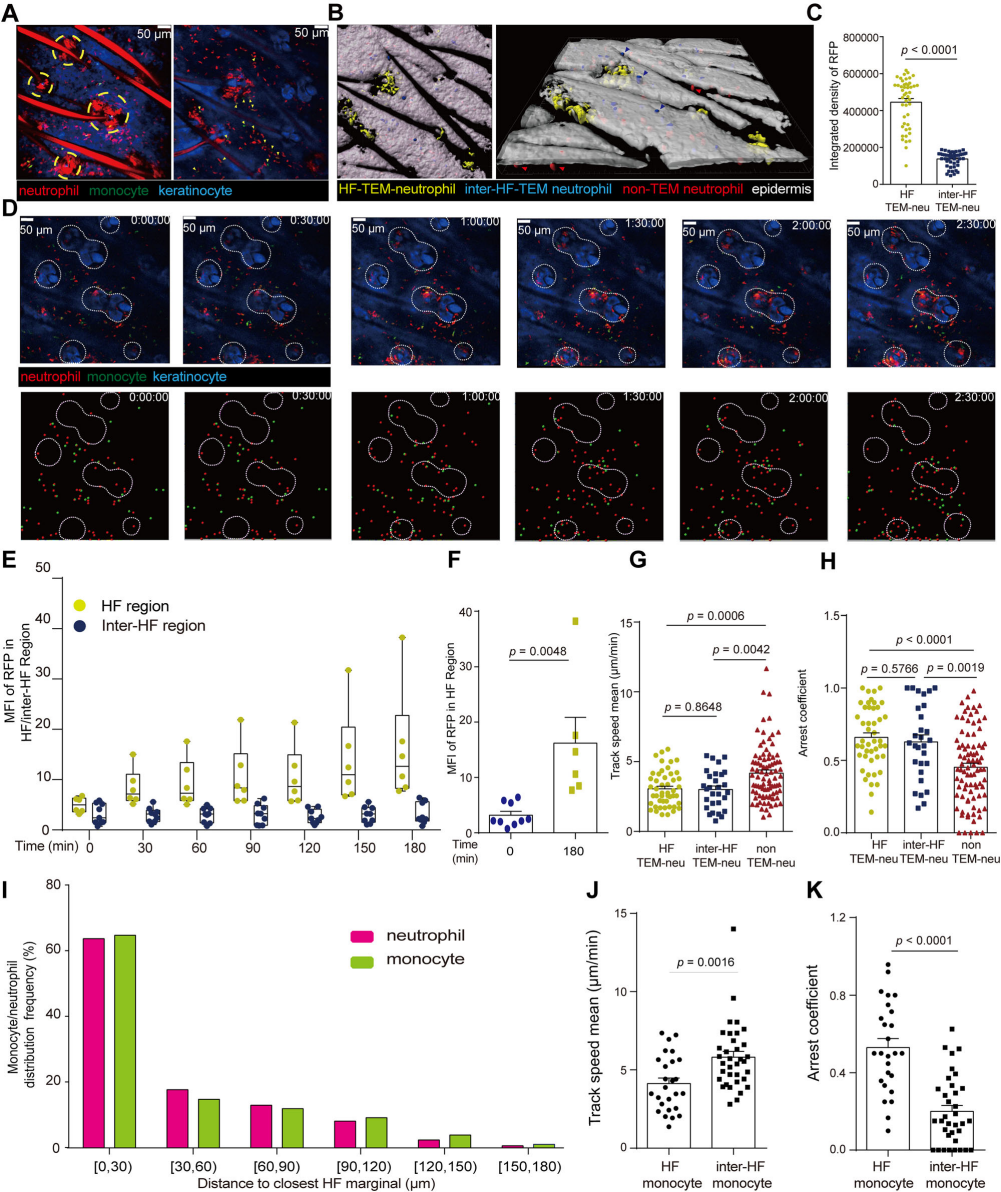
The dynamic process of NTEM in mouse ICD model. **A** Left panel: image displaying neutrophil form cluster in HF region. Yellow dashed circles indicate aggregated neutrophils. Right panel: representative neutrophil and monocyte contact image in dermis near HF region. Green arrows indicate the contacting cells. **B** Software (Imaris, Bitplane) differentiated 3 types of neutrophils with pseudo colors. HF-TEM-neutrophil (yellow), inter-HF-TEM neutrophils (blue), non-TEM neutrophils (red). **C** RFP fluorescent intensity integrated density (intDen) in HF neutrophils and inter HF TEM neutrophils, *n* = 47. **D** Representative image displaying the locations of neutrophils, monocytes, and hair follicles. Red: neutrophil, green: monocyte, blue: HF keratinocytes, white dashed circles indicating the HF region, scale bar: 50 μm. The upper parts of the images showed fluorescence-tagged neutrophils, monocytes, and keratinocytes. The lower parts were images displaying neutrophils, monocytes, and HF regions tracked by software (Imaris, Bitplane). **E** Mean fluorescent intensity (MFI) of RFP over time in HF (*n* = 6) and inter-HF regions (*n* = 9). **F** Comparison of RFP MFI in HF region of 0 min versus 180 min, *n* = 6. Mean track speed (**G**) and arrest coefficient (**H**) of HF-TEM neutrophil (*n* = 48), inter-HF-TEM neutrophil (*n* = 29), and non-TEM neutrophil (*n* = 86). **I** Frequency of neutrophils and monocytes at different distances to the closest HF marginal, *N*_(neutrophils) _= 61,822, *N*_(monocytes) _= 21,555. Mean track speed (**J**) and arrest coefficient (**K**) of monocytes in HF (*n* = 29) and inter-HF regions (*n* = 20). The arrest coefficient ranges from 0 to 1, and a value close to 1 indicates that cells have stopped moving. All the data were collected from female mice for these experiments. Data are presented as mean ± SEM from two independent experiments. Statistical analysis was performed using a two-tailed unpaired Student’s *t*-test.

Having constructed the trichromatic-fluorescence-labeled chimeric mice, we next investigated the monocyte-neutrophil crosstalk during NTEM in ICD. Here, the Krt14-EYFP signal was used to determine whether neutrophils were in the epidermis or dermis. 18 h post-OXA exposure, the intravital imaging data were collected in the Z axis at a range of 100 μm below the Krt14-EYFP signal, a region that incorporates both epidermis and dermis (Supplementary Fig. 2). The imaging data showed that epidermal neutrophils were mainly distributed in two regions, hair follicle (HF) and inter-hair follicle (inter-HF) regions (Fig. 2A, B). We compared the fluorescent intensity integrated density (intDen) of RFP in HF-TEM-neutrophils and inter-HF-TEM neutrophils, the intDen of HF-neutrophils were 3.24-fold of inter-HF-TEM neutrophils (Fig. 2C), suggesting that dermal neutrophils prefer to cross epidermis around HF (approximately 76% of TEM neutrophils). Next, we investigated the dynamic process of NTEM around the HF regions. Intravital imaging showed dermal neutrophils continuously migrated toward the HF region within 3 h (Fig. 2D, Supplementary Movie). Quantitative data confirmed that 18 h post ICD conduction, the mean fluorescent intensity (MFI) of RFP at the HF region was 1.58-fold of the inter-HF region at the beginning (Fig. 2E) and 5.07-fold of the inter-HF region at 180 min (Fig. 2F). Furthermore, TEM neutrophils displayed more restricted behavior. The migration speed of HF-TEM neutrophils and inter-HF-TEM neutrophils was 3.05 μm min^−1^ and 3.00 μm min^−1^, respectively. The arrest coefficient values were 0.659 and 0.628 in HF-TEM and inter-HF-TEM neutrophils. While the non-TEM neutrophils displayed a significantly higher migration speed (4.18 μm min^−1^) and lower arrest coefficient (value: 0.453), as seen in Fig. 2G, H. The mean square displacement versus time (MSD-T) data further confirmed that compared to non-TEM neutrophils, HF-TEM and inter-HF-TEM neutrophils were prone to more confined movement (Supplementary Fig. 3A). Similarly, monocytes showed more restricted movements in HF regions than in inter-HF regions (Supplementary Fig. 3B). Thus, in the mouse model of ICD, dermal neutrophils continually migrated toward the HF region and exhibited restricted motion at the HF region during NTEM.

Interestingly, interactions between neutrophils and monocytes were observed around HF regions. We next calculated the distance of neutrophils and monocytes to the closest HF, and the data showed that monocytes shared a similar distribution with neutrophils, in which 60.54% of neutrophils and 61.53% of monocytes were distributed with a distance to HF region below 30 μm (Fig. 2I). The monocytes near the HF region also showed more restricted movement with a lower speed (4.13 μm min^−1^) and higher arrestment coefficient (value: 0.529) than inter-HF monocytes, which had a higher speed (5.82 μm min^−1^) and lower arrestment coefficient (value: 0.200), as seen in Fig. 2J, K. These results gave hints to a close spatial relationship among neutrophils, monocytes, and HF regions during NTEM, which caused us to speculate whether the infiltrated monocytes played as gatekeepers that tune NTEM.

**Fig. 3:**
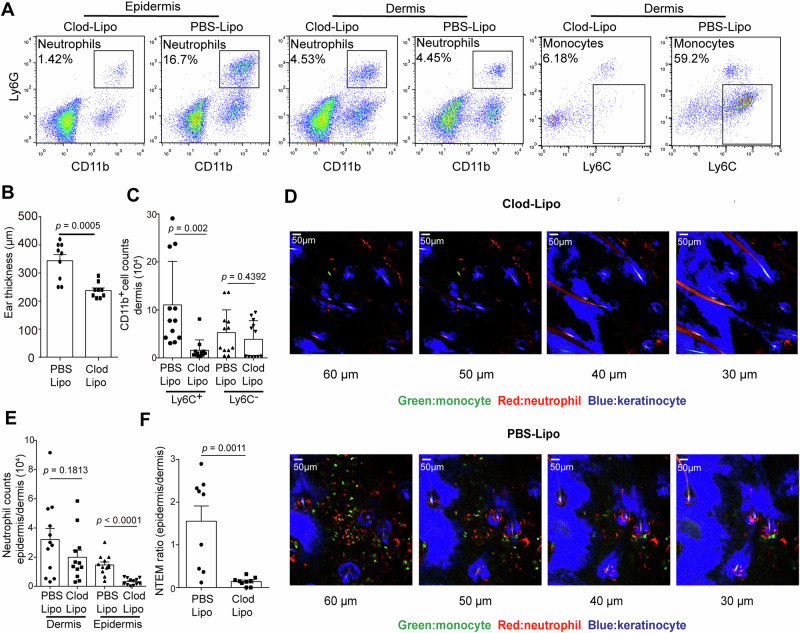
Monocytes mediate the NTEM in the skin. **A** Representative flow cytometry image displaying neutrophils and monocytes infiltration in epidermis and dermis in PBS-Lipo or Clod-Lipo-treated group. **B** Ear thickness in PBS-Lipo or Clod-Lipo-treated group (*n* = 9, means ± SEM). **C** Quantitative data of monocyte and macrophage counts in the dermis of PBS-Lipo or Clod-lipo-treated group (*n* = 12, means ± SEM). **D** Two-photo imaging of OXA-irritated chimeric reporter mouse skin in Clod-Lipo-treated (upper panel) or PBS-Lipo (lower panel) group. Image showing neutrophils and monocytes infiltration at 30, 40, 50, and 60 μm below the epidermis. Green (monocyte/GFP), Red (neutrophil/RFP), and Blue (keratinocyte/YFP). **E** Quantitative data of neutrophils counts in epidermis and dermis (*n* = 12, means ± SEM). **F** Flow cytometry analysis of epidermal/dermal infiltrated neutrophil ratio (NTEM ratio), *n* = 9, means ± SEM. All the mice were female for these experiments. Data are representative of three or more independent trials. Statistical analysis was performed using a two-tailed unpaired Student’s *t*-test.

To test this hypothesis, clodronate-liposomes (Clod-Lipo) were intravenously injected to deplete circulating monocytes 18 h before conducting ICD. The results confirmed a substantial depletion of the dermal CD11b^+^Ly6G^-^Ly6C^+^ population (mainly *Cx3cr1*^+^GFP monocytes) and decreased ear swellings in the Clod-Lipo treated group (Fig. 3A, B). As shown in Fig. 3C, the CD11b^+^Ly6G^-^Ly6C^+^ monocyte counts were significantly reduced by ~85.48% in the dermis of Clod-Lipo treated mice compared to PBS-liposome (PBS-Lipo) treated control. In contrast, no significant reduction was found in the dermal CD11b^+^Ly6G^−^Ly6C^−^ populations. These data indicated that intravenous injection of Clod-Lipo provided an effective tool for investigating the regulatory role of infiltrated monocytes in NTEM, without interfering with the CD11b^+^Ly6G^−^Ly6C^−^ populations. Next, the infiltration of neutrophils in the epidermis and dermis was calculated in monocyte-depleted and monocyte-competent ICD mice. As seen in Fig. 3D, the infiltrated monocytes and neutrophils were alleviated in Clod-Lipo-treated mice. The flow cytometry analysis (Fig. 3E) confirmed that, compared to PBS-Lipo-treated mice, neutrophil infiltration in the epidermis significantly declined from 14,640 ± 1998 to 3152 ± 659 in the Clod-Lipo-treated group. It is worth noting that neutrophil infiltration in the dermis of monocyte-depleted mice also decreased from 32,130 ± 7430 to 19,890 ± 4843 (*p* = 0.1813), suggesting that monocytes may play a role in facilitating neutrophil trans-vascular migration into the dermis, and this aspect need further exploration. To further investigate the impact of monocytes on NTEM while excluding the influence of the decrease in dermal neutrophil, we introduced the calculation of the epidermal/dermal neutrophil ratio to quantify the extent of NTEM, termed the NTEM ratio (Fig. 3F). Compared to PBS-Lipo-treated mice, the NTEM ratio was significantly decreased from 1.56 ± 0.35 to 0.14 ± 0.03, suggesting monocytes’ facilitating role in NTEM.

### MMP-9 is crucial for NTEM

**Fig. 4:**
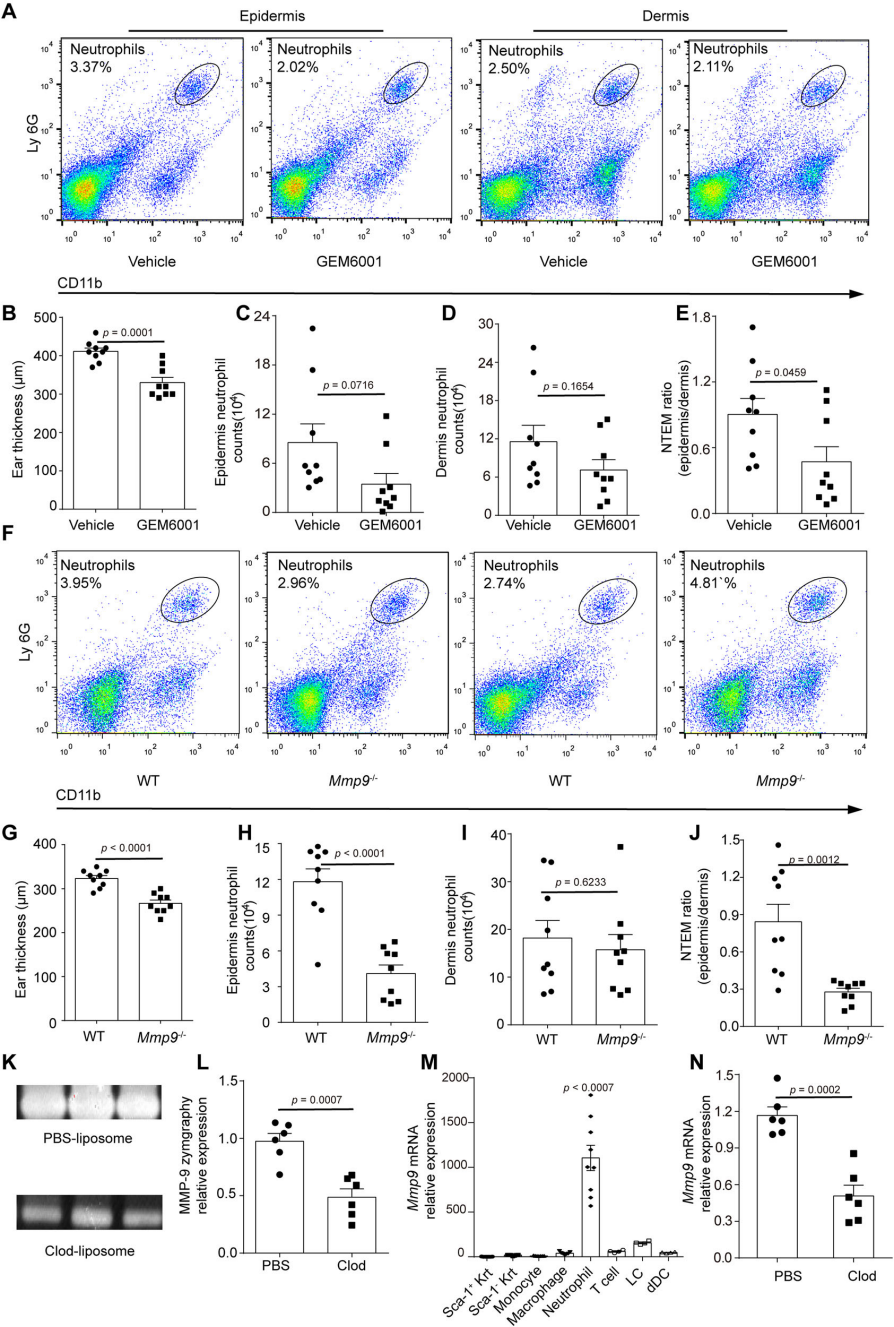
MMP-9 is crucial for NTEM. **A** Representative flow cytometry images displaying neutrophil infiltration in epidermis and dermis in MMP inhibitor or vehicle-treated group in the ICD model. **B** the ear thickness was measured 24 h post ICD conduction in the MMP inhibitor or vehicle-treated group. **C**, **D** Quantitative data of epidermal/dermal infiltrated neutrophil counts (10^4^) in A, n = 9, means ± SEM. **E** Quantitative data of epidermal/dermal infiltrated neutrophil ratio (NTEM ratio) in MMP inhibitor or vehicle-treated group. **F** Representative flow cytometry images of neutrophil infiltration in epidermis and dermis in *Mmp9*-competent and *Mmp9*-deficient mice in the ICD model. **G** The ear thickness was measured 24 h post ICD conduction in the *Mmp9-*deficient and WT mice. **H**, **I** Quantitative data of epidermal/dermal infiltrated neutrophil counts (10^4^) in (**G**) means ± SEM. **J** Statistical flow cytometry analysis of NTEM ratio in (**G**) *n* = 9, means ± SEM. **K** Upper panel: representative gelatin zymography of PBS-Lipo-treated skin lysate. Lower panel: Clod-Lipo-treated skin lysate. **L** Quantification of gelatin zymography in (**K**). *n* = 6, means ± SEM. **M** qPCR analysis of *Mmp9* mRNA expression of cells infiltrated in the dermis. *n* = 4-9, means ± SEM. **N** qPCR analysis of *Mmp9* expression in PBS-Lipo or Clod-Lipo-treated skin. *n* = 6, means ± SEM. Data are representative of two independent experiments in (**K–N**). All the mice were female for these experiments. Data are representative of three independent experiments in (**B–J**). Statistical analysis was performed using a two-tailed unpaired Student’s *t*-test in (**B**–**E**, **G**–**K**). Data in **M** were analyzed using one-way ANOVA followed by Bonferroni’s post hoc test. Bonferroni-corrected *p* values are indicated in (**M**).

The involvement of monocytes in NTEM has become clearer. We proceeded to investigate the molecular clues that may control the NTEM in the mouse ICD model. Matrix metalloproteinases (MMPs) were proposed as indicators of many pathological conditions for their role in degrading extracellular matrix components, facilitating the crossing of leukocytes through tissue membranes^28^. We first utilized a broad MMP inhibitor (GEM6001) to assess the impact of MMPs on NTEM. As expected, the administration of GEM6001 hindered NTEM. As seen in Fig. 4A, compared to vehicle-treated group, the infiltration of neutrophils in GEM6001 treated group tended to decrease in both the epidermis and dermis. The ear swelling decreased significantly in MMP inhibitor-treated group (Fig. 4B) in the ICD model. Although a clear decline trending was observed in neutrophil counts in both epidermis and dermis, no statistical difference was found between MMP inhibitor and vehicle-treated group (Fig. 4C, D). However, the NTEM ratio decreased from 0.90 ± 0.15 to 0.47 ± 0.14 (Fig. 4E). The result suggests that MMPs might be important in NTEM.

Previous studies have shown that neutrophil-secreted MMP-9 can degrade collagen and promote neutrophil migration in lungs and colorectal cancer models^29^. To investigate whether MMP-9 could modulate NTEM, we utilized *Mmp9-*deficient and *Mmp9-*competent mice to conduct ICD. As seen in Fig. 4F, compared to *Mmp9-*competent wild-type (WT) mice, *Mmp9-*deficient mice showed a significant decrease in ear swelling (Fig. 4G). A significant loss of neutrophils was observed in the epidermis of *Mmp9-*deficient mice (Fig. 4H). The dermal infiltrated neutrophils also tended to decrease, but were not significantly different with the WT mice (Fig. 4I). Interestingly, the NTEM ratio significantly declined from 0.84 ± 0.14 to 0.27 ± 0.03 (Fig. 4J). The NTEM ratio decreased by 46.67% in a broad MMP inhibitor-treated group, while the NTEM ratio decreased by 67.86% in the *Mmp9-*deficient group. Thus, the data further confirmed that MMP-9 facilitated the NTEM process in ICD.

To explore the cellular source of MMP-9 in ICD, we first analyzed the gelatin zymolytic level of MMP-9 expression in monocyte-depleted and monocyte-competent mice. A gelatin zymography was also conducted to measure the enzyme activity of MMP-9. We observed that the inflamed skin of monocyte-competent mice exhibited a higher gelatin zymolytic level of MMP-9, the secretion of MMP-9 in the monocyte-competent group was 2.26-fold of the monocyte-depleted group as confirmed by gelatin zymography (Fig. 4K, L). The cellular source of MMP-9 was mostly neutrophils (Fig. 4M) in the inflamed skin of ICD mice, as they expressed an extremely high level of *Mmp9* compared with other cells in the skin. The relative quantification (RQ) of *Mmp9* mRNA expression in neutrophils was 830.75-fold of inter-HF-keratinocytes (Sca-1^+^), 59.24-fold of HF-keratinocytes (Sca-1^−^), 106.96-fold of monocytes, 27.6-fold of macrophages, 18.38-fold of T cells, 7.06-fold of LCs and 23.2-fold of dermal DCs (dDCs) respectively. Depletion of monocyte also down-regulated the neutrophil-intrinsic *Mmp9*, the relative mRNA expression of *Mmp9* in monocyte-competent group was 2.84-fold in monocyte-depleted group (Fig. 4N). Thus, these results suggested that dermal monocytes might affect NTEM by modulating the neutrophil-intrinsic *Mmp9* expression.

### CXCL2 and TNF-α modulate NTEM

**Fig. 5:**
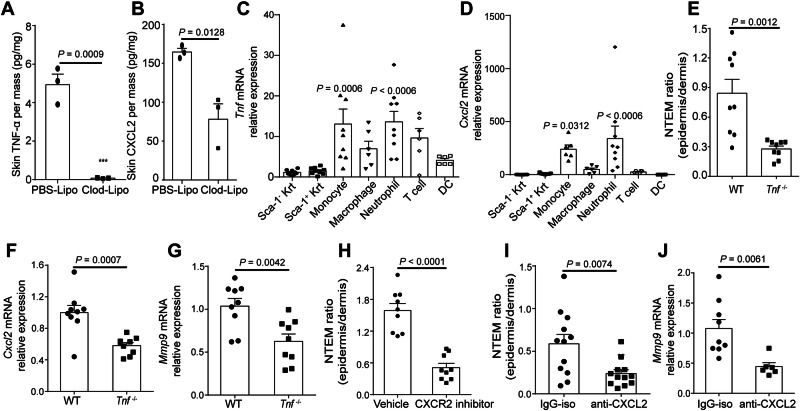
TNF-α and CXCL2 induce MMP-9 expression in neutrophils. **A** ELISA detection of skin TNF-α production in PBS-Lipo or Clod-Lipo-treated group. *n* = 3, means ± SEM. **B** ELISA detection of skin CXCL2 production in PBS-Lipo or Clod-Lipo-treated group. *n* = 3, means ± SEM. **C** qPCR examination of *Tnf* expression in skin infiltrated cells. *n* = 9, means ± SEM. **D** qPCR examination of *Cxcl2* expression in skin infiltrated cells. *n* = 9, means ± SEM. **E** Flow cytometry analysis of NTEM ratio in *Tnf*-deficient and *Tnf*-competent mice. *n* = 9, means ± SEM, 3 mice were excluded due to the technical complications. **F** qPCR analysis of *Cxcl2* expression in *Tnf*-deficient and *Tnf*-competent mice. **G** qPCR analysis of *Mmp9* expression *Tnf*-deficient and *Tnf*-competent mice. *n* = 9, means ± SEM. **H** Epidermis/dermis NTEM ratio in CXCR2 inhibitor or vehicle-treated group. *n* = 9, means ± SEM. **I** Epidermal/dermal infiltrated neutrophil ratio (NTEM ratio) in anti-CXCL2 or IgG treated group. *n* = 12, means ± SEM. **J**, qPCR analysis of *Mmp9* expression in anti-CXCL2 or IgG treated group, *n* = 6–9. Data are representative of two independent experiments in (**C**, **D**, **J**). All the mice were female for these experiments. Data are representative of three independent experiments in (**E–I**). Statistical analysis was performed using a two-tailed unpaired Student’s *t*-test in (**A**, **B**, **E–J**). Data in (**C**, **D**) were analyzed using One-way ANOVA followed by Bonferroni’s post hoc test. Bonferroni-corrected *p* values are indicated in (**C**, **D**).

It was not clear how monocytes affected neutrophil-intrinsic *Mmp9* in ICD. Previous studies have implicated cytokines in regulating MMP induction, and CXCR2 ligands have been shown to induce MMP-9^30^. In the innate immune response, proinflammatory cytokines (TNF-α, IL-1β) and neutrophil chemo-attractants (MIF, CXCL1, CXCL2, CXCL6, CXCL8) were claimed to be important for neutrophil infiltration. We then sought to identify the detailed cytokines that may regulate *Mmp9* expression in skin-infiltrated neutrophils. In the context of ICD inflammation, TNF-α and CXCL2 were detected in the skin of the monocyte-competent group, while the monocyte-depleted group exhibited reduced productions of these cytokines, as shown in Fig. 5A, B. Monocyte depletion efficiently inhibited the TNF-α and CXCL2 productions in inflamed skin, suggesting dermal monocytes were crucial for inducing or directly producing TNF-α and CXCL2 in inflamed skin of ICD mice. To further ascertain the exact cellular source of TNF-α and CXCL2 in inflamed skin, we categorized skin-infiltrated and skin-resident cells into monocytes (CD11b^+^Ly6G^−^Ly6C^+^), neutrophils (CD11b^+^Ly6G^+^Ly6C^−^), DCs (CD11c^+^MHC-II^+^), macrophages (CD11b^+^F4/80^+^), T cells (CD45^+^CD3^+^), HF-keratinocyte (CD45^−^Sca-1^−^), inter-HF-keratinocyte (CD45^-^Sca-1^+^) and purified them using flow cytometry. Interestingly, RT-PCR data showed that skin-infiltrated monocytes and neutrophils were dominant proinflammatory cells expressing *Cxcl2* and *Tnf* in the ICD model, rather than keratinocyte or other myeloid cells in the skin. Although T cells were assumed as an important source of *Tnf* in the context of many inflammation, our results showed the *Tnf* levels in T cells were not significantly different in ICD. As seen in Fig. 5C, the RQ of *Tnf* expression in monocytes and neutrophils was 13.07-fold and 13.58-fold of inter-HF-keratinocyte (Sca-1^+^) respectively. The data also showed a *Tnf* expression in T cells (Fig. 5C). However, compared to monocytes, the skin-recruited T cell proportions were relatively lower at 24 h post OXA challenge (Supplementary Fig. 4). The RQ of *Cxcl2* expression in monocytes and neutrophils were 239.99-fold and 340.17-fold of inter-HF-keratinocyte (Sca-1^+^), respectively (Fig. 5D). The relatively higher expression of *Cxcl2* and *Tnf* in skin-infiltrated monocytes and neutrophil prompted us to explore their regulatory functions on NTEM. To determine TNF-α regulatory function in NTEM, *Tnf*-deficient, and *Tnf*-competent mice were used to conduct ICD. Compared to *Tnf*-competent mice, the NTEM in *Tnf*-deficient mice was significantly inhibited, with the NTEM ratio decreasing from 0.84 ± 0.14 to 0.33 ± 0.04 (Fig. 5E). Furthermore, neutrophil-intrinsic *Cxcl2* levels in *Tnf*-competent mice were 1.69-fold *Tnf*-deficient mice (Fig. 5F). And the neutrophil-intrinsic *Mmp9* relative expression in *Tnf*-competent group were also 1.66-fold of *Tnf*-deficient group (Fig. 5G). These results confirmed the importance of TNF-α in both neutrophil-intrinsic *Cxcl2*, neutrophil-intrinsic *Mmp9* expression, and the subsequent NTEM process. Next, we explored whether CXCL2 also plays an important role in regulating NTEM. As shown in Fig. 5H, antag-CXCR2 treatment significantly inhibited NTEM, decreasing the NTEM ratio from 1.59 ± 0.13 to 0.51 ± 0.08 in the antag-CXCR2 group. We also used antibodies to block CXCL2 in vivo. Compared with the anti-IgG treated mice, anti-CXCL2 treatment effectively controlled NTEM, with the NTEM ratio decreasing from 0.59 ± 0.11 to 0.24 ± 0.04 (Fig. 5I). Additionally, the neutrophil-intrinsic *Mmp9* expression in anti-CXCL2 treated group was also significantly down-regulated, with the RQ of *Mmp9* decreasing from 1.07 ± 0.15 to 0.44 ± 0.06 when compared to anti-IgG treated group (Fig. 5J). In summary, both TNF-α and CXCL2 were important for NTEM.

### Monocyte-released TNF-α and CXCL2 facilitated neutrophil-intrinsic MMP-9 expression

**Fig. 6:**
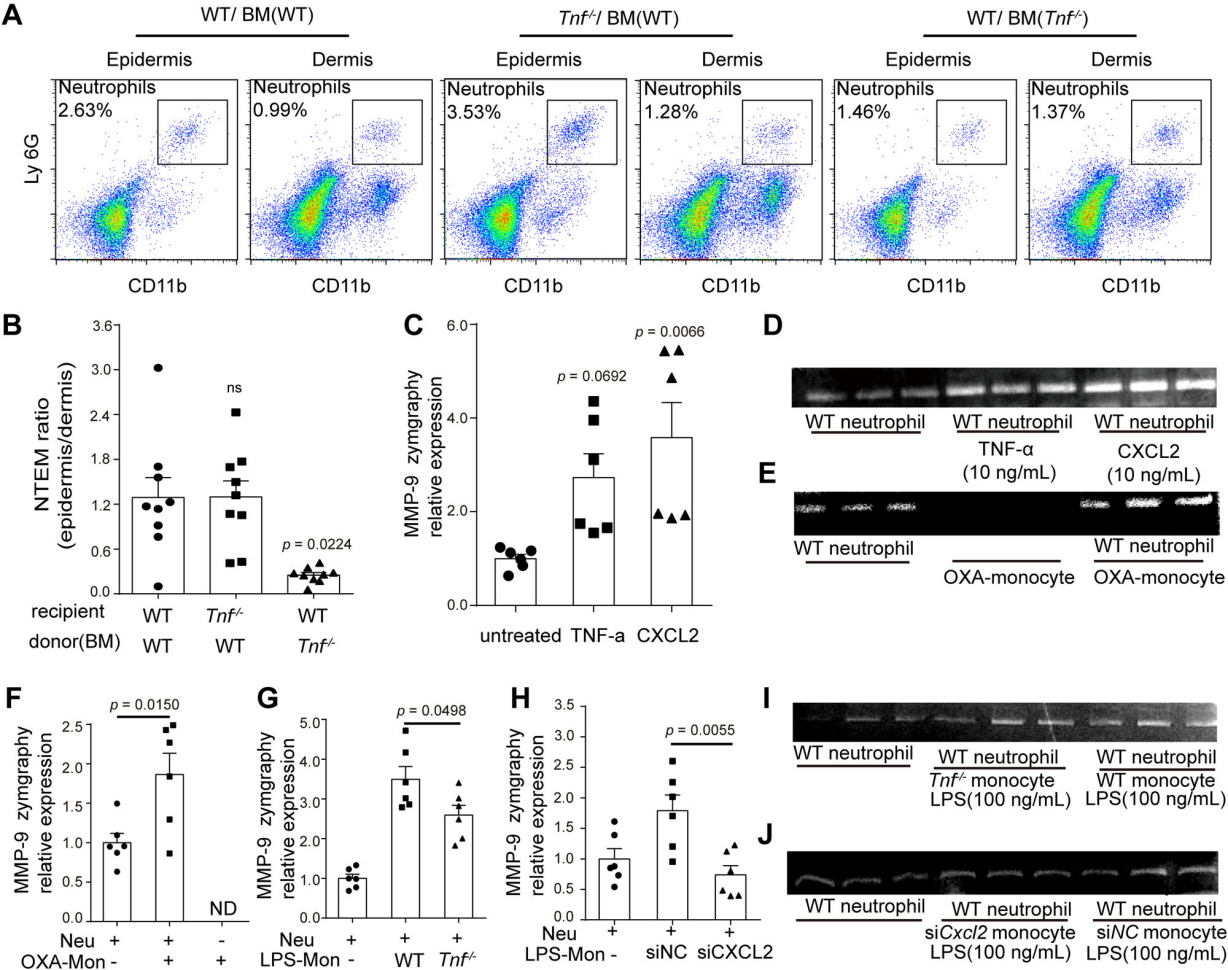
Monocytes stimulate neutrophils MMP-9 expression by releasing CXCL2 and TNF-α. **A** Representative flow cytometry images of skin infiltrated neutrophils in WT (BM WT), WT (BM *Tnf*^−/−^), and *Tnf*^-−/−^ (BM WT) groups. 6 *Tnf*-deficient male mice were used as BM donors, all the recipient mice were female. **B** Statistical flow cytometry analysis of epidermal/dermal infiltrated neutrophil ratio (NTEM ratio) in WT (BM WT), WT (BM *Tnf*^−/−^), *Tnf*^−/−^ (BM WT) group. *n* = 9, means ± SEM. **C** Quantification of gelatin zymography of invitro cultured neutrophils activated by TNF-α and CXCL2. *n* = 6, 3 male mice and 3 female mice were used to obtain BM and purify neutrophils in vitro, means ± SEM. **D** Images displaying gelatin zymography of (**C**). **E** Images display gelatin zymography of in vitro cultured neutrophils with dermal purified monocytes. **F** Quantification of gelatin zymography of in vitro cultured neutrophils with dermal purified monocytes in (**E**). *n* = 6, means ± SEM. **G** Quantification of gelatin zymography of in vitro cultured neutrophils with *Tnf*-competent-monocytes or *Tnf*-deficient-monocytes. **I** Images display gelatin zymography in (**G**). **H** Quantification of gelatin zymography of in vitro cultured neutrophils with siNC-monocytes or si*Cxcl2*-monocyte. *n* = 6, means ± SEM. **J** Images display gelatin zymography in (**H**). The monocyte in (**G**, **H**) was collected from bone marrow and stimulated with LPS before coculture with neutrophils. The gelatin zymography in (**D**, **E**, **I**, **J**) are representative images of two or three independent trials. ND, not detected, ns, no significant difference. Data in (**B**) are from three independent trials; data in (**C**, **F**, **G**, **H**) are from two independent trials. Female mice were used to obtain BM in (**E–J**). Statistical analysis was performed using a two-tailed unpaired Student’s *t*-test in (**F**, **G**, **H**). Data in (**B**, **C**) were analyzed using a one-way ANOVA followed by Bonferroni’s post hoc test. Bonferroni-corrected *p* values are indicated in (**B**, **C**).

Noticing monocyte-intrinsic *Tnf* and *Cxcl2* were upregulated in inflamed skin, global depleting monocytes or inhibiting TNF-α and CXCL2 significantly hindered NTEM as well as neutrophil-intrinsic *Mmp9* expression, we assumed that monocyte-derived CXCL2 and TNF-α were important in NTEM. Although *Tnf*-deficient mice showed impaired NTEM efficiency in ICD, *Tnf* was also reported to be broadly expressed in tissue cells, thereby inadequate to testify the regulatory role of monocyte-intrinsic *Tnf*. To distinctly testify BM-derived and peripheral tissue-derived TNF-α function, we first utilized *Tnf*-deficient bone marrow as donors and *Tnf*-competent mice as recipients to build BM chimeric mice, or conversely, *Tnf*-competent BM were adoptively transferred to *Tnf*-deficient mice. *Tnf*-competent BM cells were also trans-adopted to other *Tnf*-competent mice as a control to exclude the impact of irradiation. Then we explored the NTEM behavior in chimeric mice. The NTEM ratios were 1.29 ± 0.27, 1.30 ± 0.22, and 0.25 ± 0.03 in WT (BM WT), *Tnf*-deficient (BM WT), and WT (BM *Tnf*-deficient) respectively. The NTEM ratio were significantly inhibited only in BM *Tnf*-deficient group (Fig. 6A, B), confirming that BM-derived TNF-α was important in NTEM.

To determine the regulatory roles of TNF-α and CXCL2 in MMP-9, we next tested the MMP-9 gelatin zymolytic level in an in vitro coculture system. Recombinant TNF-α and CXCL2 were used to stimulate purified BM neutrophils. CXCL2 significantly upregulated the MMP-9 level as seen in Fig. 6C, D. At the same time, TNF-α stimulation showed a slightly up-regulated MMP-9 zymolytic level, but the difference was not statistically significant. The secreted MMP-9 in CXCL2-stimulated neutrophils were 1.31-fold of the TNF-α-stimulated group and 3.58-fold of the non-stimulated group, as gelatin zymography result proved. Next, we isolated the skin-infiltrated inflammatory monocyte from OXA-challenged ICD mice and cocultured them with purified BM neutrophil. The gelatin zymography data in Fig. 6E confirmed that skin-infiltrated inflammatory monocytes increased neutrophil-released MMP-9. Consistent with the qPCR detection of cell intrinsic-*Mmp9* in Fig. 4M, the secreted MMP-9 in inflammatory monocytes was also not detected in vitro. After coculturing with activated monocytes purified from hapten-challenged skin, the MMP-9 zymolytic level in the monocyte-neutrophil coculture system was significantly increased, as the MMP-9 level was 1.84-fold of the non-coculture group (Fig. 6F). The results supported that activated monocytes effectively promote MMP-9 secretion in neutrophils. To explore whether inflammatory monocyte-derived TNF-α and CXCL2 were the key molecules in regulating NTEM, *Tnf*-deficient monocytes, *Cxcl2* knock-down monocytes, and wild-type (WT) neutrophils were purified and harvested for the in vitro coculture system. Interestingly, the ability to stimulate MMP-9 secretion was reduced in the *Tnf*-deficient monocyte coculture group. The MMP-9 secretion in the coculture system of WT neutrophils and LPS-stimulated *Tnf*-competent monocytes was 3.49-fold of the non-coculture group. While in the coculture system of WT neutrophils and LPS-stimulated *Tnf*-deficient monocyte, the MMP-9 secretion was 2.59-fold of the non-coculture group (Fig. 6G, I). Moreover, the knock-down of *Cxcl2* in monocytes also led to lower MMP-9 level, the MMP-9 secretion in the *Cxcl2*-competent monocytes treated group was significantly decreased, the MMP-9 zymolytic level in the *Cxcl2*-competent group was 2.35-fold of *Cxcl2* knock-down group (Fig. 6H, J). Considering neutrophils also expressed high levels of *Tnf* and *Cxcl2* in the OXA-induced ICD model (Fig. 5C, D), we next examined whether neutrophils could amplify the production of MMP-9 by autocrine pattern. Purified neutrophils from OXA-treated skin in CD45.2^+^ mice were incubated with untreated neutrophils sorted from the bone marrow of CD45.1^+^ mice. As shown in Supplementary Fig. 5, the MFI of MMP-9 in the cocultured system was 2.94-fold of the untreated neutrophils, implying the autocrine production of TNF-α and CXCL2 by neutrophils can amplify the production of MMP-9. These results suggested that in the OXA-induced ICD, the skin-infiltrated monocytes and neutrophils were both essential in triggering neutrophil-secreted MMP-9 and mediating NTEM.

## Discussion

Increasingly clinical studies have found that the occurrence and development of inflammatory skin diseases are accompanied by epidermal infiltration of neutrophils, especially in patients with atopic dermatitis or pustular psoriasis^31–33^. Neutropenia can significantly alleviate the inflammatory symptoms of atopic dermatitis^13^, allergic contact dermatitis^14^, and psoriasis^34^. Previously, researchers have focused on the process of neutrophils and monocytes trans-endothelial migration^18,35–39^, while how neutrophils transmigrate from the dermis to the epidermis and the monocyte-neutrophil interaction in dermis is unclear.

Intravital imaging has provided a powerful tool to study the motility, morphology, and activation of immune cells in vivo. It is of great value to simultaneously monitor neutrophil, monocyte, and skin structure in vivo through multi-color labeling of immune cells involved in inflammatory skin disorders. However, there are no available mouse strains that simultaneously track monocytes, neutrophils, and keratinocytes in the skin. Our approach, using *Cx3cr1*^GFP/+^*Lyz2*^RFP/+^ mice as bone marrow (BM) donors and Krt14^YFP/+^ mice as recipients to reconstitute the trichromatic-fluorescence labeled chimeric mice, simultaneously visualize the monocytes, neutrophil, and keratinocyte in the skin with high labeling accuracy (over 90%) at 2-4 weeks post BM reconstitution. In our study, we successfully reconstructed chimeric mice with different fluorescent proteins (GFP, EYFP, RFP) and high labeling accuracy (over 90%) to simultaneously distinguish epidermal keratinocytes, neutrophils, and monocytes. One limitation of the approach is the reconstitution of dermal macrophages by *Cx3cr1*^GFP/+^*Lyz2*^RFP/+^ bone marrow at 6 weeks after the BM chimeric experiment. The expression of RFP and the loss of GFP in dermal macrophages make it difficult to differentiate between neutrophils and dermal macrophages. Imaging of neutrophil-monocyte-keratinocyte crosstalk should be conducted within 3–6 weeks after X-ray irradiation; however, this time window is sufficient for us to complete the imaging work monitoring neutrophil migration across the epidermis during mouse skin inflammation.

Taking advantage of intravital imaging, we observed neutrophils trans-epidermal via HF regions, while monocytes were rarely seen transmigrated from dermis to epidermis (Supplementary Fig. 6). Site preference of neutrophil transmigration during epithelial inflammation has also been reported in other epithelial regions. For instance, neutrophil transepithelial migration in the intestines was found to be restricted to submucosal vessels in response to salmonella/endotoxin-induced inflammation, with elevated levels of CXCL1 and CXCL2 observed in the submucosa laye^40^. Recent studies suggest that hair follicles play a crucial role in immune cell recruitment, including Langerhans cells, monocytes, and T cells, and act as both sites for cutaneous immune responses and immune privilege^41^. In our result, monocytes were also observed recruited around HF region in the ICD model. One possibility is that the presence of monocytes around the hair follicles may activate nearby neutrophils, prompting them to express higher levels of MMP-9. Nagao K et al. reported that HF-keratinocyte subsets were a chief source of chemokines able to promote the recruitment of LCs^42–44^. Another possibility is that the HF-keratinocytes might express a higher level of chemokines such as CXCL1 and CXCL2 to chemo neutrophils accumulation and subsequently assist NTEM in HF regions. These hypotheses will require further experimental investigation to validate. It is also not clear if neutrophils adopt the same motility pattern as LCs when transmigrating from the dermis to the epidermis. Thus, an intravital imaging study of the neutrophil transmigrating pattern is urgently needed.

More and more researchers have begun to recognize the role of monocytes, macrophages, and mast cells in regulating neutrophil migration. For instance, mast cells and macrophages are involved in the control of IL-1β-induced neutrophil migration in rats^45^. Macrophage migration inhibitory factors (MIF) were reported to inhibit human blood-sorted neutrophil apoptosis, and the effect involves the activation of mononuclear cells, which secrete CXCL8 and other prosurvival mediators to promote neutrophil survival^46^. Infiltrated inflammatory monocytes are also reported to play a key role in the clearance of highly motile apoptotic neutrophils during the resolution phase in infected mouse airway^47^. In aged liver, CXCL2^+^ macrophages presented a senescence-associated secretory phenotype and recruited neutrophils to aggravate liver injury through the secretion of IL-1β and TNF-α^48^. A study of urinary tract bacterial infection found that the bone marrow-derived Ly6C^+^ monocyte infiltrates the bladder and cooperates with bladder-resident Ly6C^−^ macrophages to regulate neutrophils recruitment from blood and eventually to uroepithelium^30^. However, there are great differences among skin, airway, gastrointestinal mucosa, liver, and urinary tract systems. Whether monocytes were involved in modulating neutrophil migration across the epidermis in innate immunity is still unclear. In our results, dermal neutrophils dynamically contacted Ly6C^+^CX3CR1^+^ dermal monocytes around the HF region and preferred to trans-migrate into the epidermis in this area. The major participants were Ly6C^+^ monocytes rather than Ly6C^−^ monocytes in OXA-induced ICD (Supplementary Fig. 7). Dermal infiltrated Ly6C^+^ monocytes not only express TNF-α but also directly express high-level CXCL2 to mobilize NTEM in contact dermatitis. Ly6C^hi^ monocytes, other than structural or environmental cells in the dermis, acted as gatekeepers that released CXCL2 and TNF-α to upregulate MMP-9 expression in neutrophil, and eventually mediated NTEM.

CXCL2 is a growth-related oncogene and chemotactic factor of neutrophils, produced by activated monocytes and macrophages^49^. A study reported that epidermal keratinocytes can also produce CXCL2 and recruit neutrophils to skin lesions of patients with psoriasis and its animal models^50^. Further, keratinocytes secret MMP-1, a collagenase that degrades fibrillar collagens type I and III into specific fragments, and they also produce MMP-9, which digests collagen type IV, an important component of skin basement membrane^51–54^. However, our data showed both Sca-1^+^ or Sca-1^-^ keratinocytes expressed low levels of *Mmp9*, *Tnf*, and *Cxcl2* at 24 h post OXA exposure. MMP-9 has also been associated with Langerhans cell migration. However, in OXA-induced ICD, the LCs’ *Mmp9* levels were significantly lower than neutrophils (Fig. 4M). In the future, LC-depletion or conditionally knock out MMP-9 in LC is needed to study its possible contribution in the early phase of NTEM. An early expression of thymic stromal lymphopoietin (TSLP) at 4 h was reported in keratinocytes of skin irritation, TSLP is also a key driver for the downstream inflammatory cascade^55^. We examined the *Tslp* mRNA expression in keratinocytes at 4 and 24 h post-OXA challenge. The *Tslp* relative expression in Sca-1^+^ keratinocytes showed a clear elevating trend at 24 h post-OXA-challenge compared to 4 h, but no significant differences were found when using an unpaired Student’s *t*-test. While in Sca-1^−^ keratinocytes, the *Tslp* significantly downregulated at 24 h compared to 4 h (Supplementary Fig. 8). Perhaps the upregulation in Sca-1^+^ keratinocytes at early stage of ICD plays an important role in initiating NTEM process. Whether the early expression of *Tslp* and its downstream inflammatory cytokines plays a role in the context of NTEM process of ICD needs further investigation.

Neutrophils are an important source of TNF-α and CXCL2 in some inflammatory conditions^56^. Like other myeloid lineage cells, neutrophils produce TNF under a variety of settings, including M. tuberculosis infection and air pouch model of inflammation^57,58^. T cells were also an important source of TNF in allergy and autoimmune disease, in which adaptive immune response plays a major role. In the context of OXA-induced ICD, our data showed a *Tnf* expression in T cells (Fig. 5C). However, compared to monocytes, the skin-recruited T cell proportions were relatively lower at 24 h post OXA challenge. Further investigations are needed to study the contribution of T cell-derived *Tnf* in the NTEM process. In cytokine-stimulated mouse cremaster muscles, neutrophils were the main producers of CXCL2, which was critical for neutrophils to breach endothelial junctions^59^. In our study, MMP-9 and CXCL2 were proved to be crucial in NTEM, CXCL2 was effective in activating neutrophil-intrinsic *Mmp9* expression, and MMP-9 participated in basement membrane degradation which eventually allowed neutrophil transmigration. Yet our study showed keratinocytes or skin-resident macrophages express relatively low levels of those cytokines in ICD, the main cellular source of *Tnf* and *Cxcl2* were neutrophils and monocytes, and neutrophils were dominant MMP-9 producers (Figs. 4M, and 5C, D). These results suggested that the monocytes might act as gatekeepers that controlled NTEM.

One limitation of the study is that we didn’t test the role of mast cells or fibroblasts in the NTEM process. Some studies have reported mast cells might produce TNF and histamine to activate DCs in mouse models of allergic dermatitis^60,61^. In parallel, mast cells were also aided by skin macrophages to recruit neutrophils from the periphery^62^. Whether mast cells or fibroblasts also participate in recruiting neutrophils in irritating dermatitis remains further investigated.

To conclude, we developed a multi-fluorescence, endogenous labeled transgenic mouse model to study neutrophil-monocyte-keratinocyte crosstalk in vivo. Our study characterized a monocyte-mobilized NTEM behavior in ICD. Monocyte-derived cytokines TNF-α and CXCL2 were important molecules to stimulate neutrophils. Subsequently, activated neutrophils secrete MMP-9 to degrade the collagen, allowing NTEM. Our study offers intuitive evidence for how dermal monocyte modulates NTEM in a pathological skin environment, thereby providing a distinct perspective on the treatment of inflammatory skin disorders through the targeting of monocyte-mediated neutrophil infiltration into the epidermis.

## Methods

### Mice

Mice were housed in cages (≤5 mice) with free access to food and water in the specific pathogen-free barrier facility at Wuhan National Laboratory for Optoelectronics (WNLO) ’s Animal Center, in a temperature- and humidity-controlled room (23 ± 3 °C, 50 ± 20% RH) under a 12-h light period, 12-h dark period (lights on from 8:00 to 20:00) cycle. C57BL/6 J (MGI: 5656552, Strain:000664) female mice were supplied by the Hubei Research Center of Laboratory Animals (Hubei, China). *Cx3cr1-*GFP mice: B6.129P-*Cx3cr1*^*tm1Litt*^/J (Strain:005582); *Tnf-*deficient mice: B6.129S-*Tnf*^*tm1Gkl*^/J (Strain:005540); *Mmp9-*deficient mice: B6.FVB(Cg)-*Mmp9*^*tm1Tvu*^/J (Strain:007084); *Lyz2*-cre mice: B6.129P2-*Lyz2*^*tm1(cre)Ifo*^/J (Strain:004781); *R26R*-EYFP mice: B6.129×1-*Gt(ROSA)26Sor*^*tm1(EYFP)Cos*^/J (Strain:006148); Ai14 mice: B6.Cg-*Gt(ROSA)26Sor*^*tm14(CAG-tdTomato)Hze*^/J (Strain:007914); K14-cre mouse: Tg(KRT14-cre)1Amc/J (Strain:004782); and B6 CD45.1 mice: B6.SJL-*Ptprc*^a^ *Pepc*^b^/BoyJ (Strain #:002014) were obtained from Jackson Laboratory (Bar Harbor, Maine, USA). For breeding schemes: *Lyz2*-RFP heterozygote mice were obtained by crossing *Lyz2*-cre homozygous mice and Ai14 homozygous mice, Krt14-EYFP heterozygote mice were obtained by crossing K14-cre homozygous mice with *R26R*-EYFP homozygous mice. *Lyz2*^RFP/+^*Cx3cr1*^GFP/+^ were obtained by crossing *Cx3cr1*-GFP homozygous mice with *Lyz2*-RFP heterozygote mice. All animal studies were performed under the Experimental Animal Management Ordinance of Hubei Province, P.R. China, and were approved by the Institutional Animal Care and Use Committee at Tongji Medical College, Huazhong University of Science and Technology (IACUC Number: 844). All experimental procedures involving live vertebrates were conducted in accordance with relevant guidelines and regulations. We have complied with all relevant ethical regulations for animal use.

### Induction of mouse irritant contact dermatitis (ICD) models

Female C57BL/6J mice, aged 6–8 weeks and weighing 20–22 g, were used to conduct ICD. Mice were anesthetized with an intraperitoneal injection of a mixture of 10 mg/kg xylazine and 100 mg/kg ketamine hydrochloride (Sigma, USA). After the breath and pulse were stabilized, the periauricular hair of mice was trimmed. Subsequently, the dorsal and ventral sides of the ear were painted with 10 μL of 1% (w/v) oxazolone (Cat.No. 862207, Sigma, USA) diluted in a vehicle (4:1 acetone: olive oil). After stimulation for 24 h, the change in ear thickness was measured using a micrometer (Mitutoyo) to assess the ear swelling.

### Reconstitution of bone marrow chimeric mice

*Lyz2*^RFP/+^*Cx3cr1*^GFP/+^ heterozygote mice, *Tnf-*deficient homozygous mice, and C57BL/6 J mice were used as bone marrow donors. Bone marrow cells were harvested from the forelimbs and hindlimb tibias of mice using a 1 mL syringe. The collected BM was resuspended and homogenized in RPMI 1640 medium (Hyclone, USA). Red blood cells were lysed using ACK lysis buffer, and the remaining cells were filtered through a sterile 70 μm cell strainer to remove debris. The filtered bone marrow cells were washed twice with cold PBS, resuspended, and kept on ice for subsequent use. Female Krt14-YFP heterozygote mice and C57BL/6 J mice were used as recipients, the recipients were irradiated with a single dose of 9 Gy by 6-MV x-rays (600 MU/min, Trilogy System LinearAccelerator, Varian Medical Systems) to clear the bone marrow. To delay the reconstitution of RFP^+^GFP^−^ dermal cells, the ear of the Krt14-EYFP mouse was shielded with a 5 mm lead plate to protect the original recipient mouse dermal DCs/macrophages from X-ray irradiation. Two hours following myeloablation, recipient mice were intravenously injected with 5 × 10^6^ bone marrow cells. Mice were fed with antibiotic water containing 2 mg/mL neomycin sulfate (Cat.No. N412785, Aladdin, China) and 100 U/mL polymyxin B sulfate (Cat.No. P105490, Aladdin, China) for two weeks after irradiation to prevent infection.

### Intravital imaging of inflammatory monocytes, neutrophils, and keratinocytes

The chimeric mice were anesthetized using a mixture of 50% oxygen and 1–3% isoflurane (Cat.No. R510-22-10, RWD Life Science, China) for 2–6 h. After the animals’ respiration and heart rate stabilized, and the absence of corneal reflex, tactile response, and pinch reflex was confirmed, the mice were imaged using a LSM 780 NLO multiphoton confocal microscope (Zeiss, Germany) equipped with either a 10× (NA 0.45) or a 20× (NA 1.0) water immersion objective. The body and ear of the mice were placed on separate temperature maintenance devices (RWD Life Science, China) to ensure their physical signs. For capturing the interstitial migration of monocytes and neutrophils, images were acquired 18 h post OXA exposure at a rate of two frames per minute and a z-step of 10 µm in a range of 100 μm. For simultaneously capturing the movement of monocyte, neutrophil, and keratinocyte in vivo, Krt14-YFP (bone marrow, *Cx3cr1*^GFP/+^/*Lyz2*^RFP/+^) chimeric mice were used to induce ICD. GFP-labeled monocytes, RFP-labeled neutrophils, and YFP-labeled keratinocytes were imaged in the ICD-inducing mice. Images were acquired at a rate of six frames per minute and a z-step of 10 µm. The excitation wavelengths of GFP and EYFP were excited with a 488 nm femtosecond pulsed laser, and RFP was excited with a 561 nm femtosecond pulsed laser. Among them, the GFP and YFP signal excitation crosstalk was separated by spectral unmixing and lambda linear unmixing using Zeiss Zen microscopy software. With the above settings, the image size was 424 μm × 424 μm, and the resolution was 512 × 512 pixels. Using Imaris 7.2.1 software (Bitplane), we implemented the realization of three-dimensional reconstruction, the calculation of monocyte/neutrophil displacement, mean speed, confinement ratio, and arrest coefficient, as well as the processing of movies. To define the HF and inter-HF neutrophils, we used the whole imaging region as an “intact cell”, Krt14-EYFP signal in the HF region was utilized to mimic the nucleus and *Lyz2*-RFP signal was utilized to mimic the “vesicles”; the imaris algorithm in the “cell” module was then applied to semiautomatically define the HF and inter-HF neutrophils. The non-TEM and inter-HF-TEM neutrophils were tracked manually.

### Epidermis and dermis separation for cell isolation

The mouse ears were collected 24 h post-OXA exposure, mice were culled by cervical dislocation under deep anesthesia induced by intraperitoneal injection of a mixture of 10 mg/kg xylazine and 100 mg/kg ketamine hydrochloride. To obtain the intact skin from the mouse ear, carefully dissect along the edges of the ear using scissors and fine-tip forceps. Ensure that the entire piece of skin is removed without damage. The inflamed ears were divided into dorsal and ventral parts with fine-tip forceps, carefully removing the cartilage of skin via fine-tip curved forceps. The dissected skin samples were digested in PBS (Hyclone, USA) containing 5 U/mL Dispase II (Cat.No. 04942078001, Roche, Switzerland) in a 6-well plate at 37 °C in a water bath for 1.5 h. The epidermis and dermis were separated using forceps and digested in 1640 containing 0.33 mg/mL Liberase TL (Cat.No. 5401020001, Roche, Switzerland) at 37 °C for 1.5 h. The digested tissue was gently dissociated using a 1 mL pipette until dermis could be easily dispersed. The digested skin tissues were then filtered using a 70 μm nylon mesh to obtain single-cell suspension. The cell suspension was washed twice with fluorescence-activated cell sorting (FACS) buffer, which consists of PBS; supplemented with 2% fetal bovine serum (FBS; Gibico, USA) and 1 mM ethylenediaminetetraacetic acid (EDTA; Sinopharm, China), and 1% penicillin-streptomycin (Gibico, USA).

### Flow cytometry

Before staining, the single-cell suspension was blocked with anti-mouse FcRII/III (clone:2.4G2, Cat. No. 553141) at 4 °C for 10 min. The staining was done in the FACS buffer at 4 °C for 30 min using the following antibodies: anti-mouse antibodies specific for CD45 (clone:30-F11, Cat. No. 557659), Ly6C (clone: HK1.4.rMAb, Cat. No. 569434), Ly6G (clone: 1A8, Cat. No. 551461), and CD11b (clone: M1/70, Cat. No. 552805) were purchased from BD Bioscience (CA, USA). Anti-mouse CD192 (clone: SA203G11, Cat. No. 150605) was purchased from Biolegend (CA, USA). The dead cells were excluded with PI (Cat. No. 550825, BD Bioscience) or dye eFluor^TM^ 780 (Cat. No. 65-0865-14, Thermo Fisher). For intracellular staining, cells were initially stained for surface markers, followed by fixation and permeabilization at 4 °C for 20 min using Fixation Buffer (Cat. No. 420801, Biolegend, USA). After washing twice with diluted Intracellular Staining Permeabilization Wash Buffer (Cat. No. 421002, Biolegend, USA), cells were incubated with MMP-9 recombinant antibody (clone: 230069F1, Cat. No. 82854-5-RR, Proteintech, China) for 2 h, and then stained with multi-rAb™ CoraLite Plus 647-Goat anti-rabbit recombinant secondary antibody (H + L, Cat. No. RGAR005, Proteintech, China) for 1 h. Flow cytometry antibodies (0.5 mg/mL or 0.2 mg/mL) were diluted 1:500 or 1:200 in staining buffer. After staining, cells were washed twice with PBS and analyzed by flow cytometry. Data acquisition was performed using a Guava InCyte analyzer (Guava Technologies, CA, USA) or CytoFLEX flow cytometer (Beckman Coulter, CA, USA). Fluorescence-activated cell sorting was conducted on a Moflo XDP cell sorter (Beckman Coulter, CA, USA). Data analysis was carried out using FlowJo software (Ashland, USA). The gating strategy for FACS plots is illustrated in Supplementary Fig. 9.

### Statistics analysis of absolute cell counts and NTEM ratio calculation

The absolute cell counts in the epidermis and dermis were counted using a Guava ViaCount analyzer. In brief, the cell suspension of the epidermis and dermis were stained with ViaCount buffer (Guava Technologies, CA, USA) to calculate the live cell counts of each part, the neutrophil counts were calculated by whole cell counts of epidermis or dermis multiplied by the CD11b^+^Ly6G^+^Ly6C^−^ percentages in each part. The NTEM ratio was calculated using the absolute counts of epidermal infiltrated neutrophils divided by dermal infiltrated neutrophils.

### Monocyte depletion and inhibition of NTEM

To deplete monocytes, female mice (weighing ~20 g) were intravenously injected with 200 μL of Clod-Lipo (Cat. No. CP-005-005, BIOHUB, China) or PBS-Lipo as a control. Prior to injection, Clod-Lipo and PBS-Lipo were thoroughly vortexed to ensure homogeneity. Monocyte depletion was initiated 18 h before ICD induction, and the depletion efficiency was assessed in blood and skin samples 24 h post-injection. To investigate the role of CXCR2 in NTEM, CXCR2 inhibitor SB225002 (Selleck, USA) was dissolved in a vehicle (5% DMSO, 40% PEG300, 5% Tween 80, 50% ddH2O) according to the manufacturer’s instructions. Female mice (weighing ~20 g) were administered SB225002 intraperitoneally at a dose of 5 mg/kg three times within 24 h during ICD induction. To study the function of CXCL2 in NTEM, female mice (weighing ~20 g) were intravenously injected with 30 μg of anti-mouse CXCL2 monoclonal antibody (Cat. No. MAB452-500, R&D Systems, USA) or an isotype control (Rat IgG2b, clone: 141945, Cat. No. MAB0061, R&D Systems, USA) 6 h before ICD induction.

### Quantitative PCR analysis

The skin tissue was snap-frozen in liquid nitrogen, homogenized with TRIZOL™ reagent (Cat. No. 15596026CN, Thermo Fisher Scientific, USA), and stored at −80 °C until further use. Skin cells were isolated and purified through fluorescence-activated cell sorting (FACS). Total RNA from the skin tissue and sorted cells were extracted using TRIZOL™ reagent, with glycogen (Cat. No. R0561, Thermo Fisher Scientific, USA) employed as a carrier. The RNA extraction process was performed strictly in accordance with the manufacturer’s protocol. Quantification and quality assessment of the RNA using a Bio-Photometer (Eppendorf, Germany). The PrimeScript RT Mast Mix kit (Cat. No. RR036A, TaKaRa, Japan) was used to reverse transcribe RNA into cDNA according to the manufacturer’s protocol. Using SYBR Premix Ex Taq^TM^ II (Cat. No. RR390A, TaKaRa, Japan) for subsequent quantitative PCR, which was performed with Applied Biosystems Stepone Detector (Applied Biosystems, USA). The sequences of the primers are shown below:

*Actb* forward, 5’- GGCTGTATTCCCCTCCATCG-3’.

reverse, 5’- CCAGTTGGTAACAAT GCCATGT-3’.

*Cxcl2* forward, 5’-CCAACCACCAGGCTACAGG-3’.

reverse, 5’-GCGTCACACTCAAGCTCTG-3’.

*Tnf* forward, 5’-CCCTCACACTCAGATCATCTTCT-3’.

reverse, 5’-GCTACGACGTGGGCTACAG-3’.

*Mmp9* forward, 5’-CTGGACAGCCAGACACTAAAG -3’.

reverse, 5’-CTCGCGGCAAGTCTTCAGAG-3’.

*Tslp* forward, 5’-ACGGATGGGGCTAACTTACAA -3’.

reverse, 5’-AGTCCTCGATTTGCTCGAACT-3’.

### Gelatin zymography

Gelatin zymography was used to measure the enzyme activity of MMP-9 in the skin and purified neutrophils. Supernatants in neutrophil (1 × 10^5^) and monocytes (2 × 10^4^) coculture systems were collected to examine the secreted MMP-9’s gelatin zymolytic level. Gelatin zymography protocols were performed as described previously with some modification^63^. Briefly, preparing 8% SDS-page separating gel with 0.5–1% gelatin and 5% SDS-page stacking gel, proteins were loaded with a non-denaturing loading buffer and separated at 125 V for 2 h. The gel was then incubated for 1 h at room temperature in a renaturation buffer with the following formulation: 50 mM Tris-HCL (pH 7.5), 25 mM CaCl_2_, 1 μM ZnCl_2_, TritonX-100 2.5% (v/v). Subsequently, wash the gel twice for 5 min in a Washing buffer with the following formulation: Tris-HCL (pH 7.5), 25 mM CaCl_2_, 1 μM ZnCl_2_. Incubated the gel for 48 h at 37 °C in development buffer with the following formulation: Tris-HCL (pH 7.5), 25 mM CaCl_2_, 1 μM ZnCl_2_, NaCl 25 mM, and 0.2% Brij-35. After 45 min of staining with 0.5% Coomassie, the gel was incubated for 30 min using decolorization buffer with the following formulation: 50% Methanol, 40% Aqua, and 10% Acetic acid. Chemicals were obtained from Macklin Biochemical Co., Ltd, China. Finally, the digital analysis of the gel was achieved by a Gel Doc XR^+^ Documentation System (BioRad, USA), and the density of the band was quantified using ImageJ software.

### ELISA for detecting the expression of TNF-α, CXCL2

To prepare homogenates from mouse ear skin, the tissue was finely minced and incubated in a lysis buffer consisting of PBS, 1% Triton X-100, and a protease inhibitor cocktail (Cat. No. P840, Sigma, USA). The minced tissue was homogenized in a 5 mL round-bottom tube using an electric homogenizer on ice. The resulting homogenate was filtered through a 0.45 μm membrane and stored at −80°C until use. TNF-α (Cat. No. 430904, BioLegend, USA) and CXCL2 (Cat. No. MM200, R&D, USA) ELISA kits was used to analyze the cytokine production in PBS-Lipo-treated skin and Clod-Lipo-treated skin. ELISA was performed according to the manufacturer’s manual. The O.D. of the samples was detected with a UV–visible absorbance microplate reader (FlexStation, USA) at a wavelength of 450 nm.

### Incubation of neutrophils and monocytes in vitro

FACS and magnetic-activated cell sorting (MACS) were used to purify monocytes and neutrophils for coincubation in vitro. In brief, naïve neutrophils were obtained from BM and purified to 90% using EasySep™ Mouse Neutrophil Enrichment Kit (Cat. No. 19762, Stemcell technology, Canada) and were incubated 1 × 10^5^ per well in 96-well flat-bottom plates. LPS pretreated BM monocytes were enriched to >90% purity using EasySep™ Mouse Monocyte Isolation Kit (Cat. No. 19861, Stemcell technology, Canada) and OXA-treated inflamed skin monocytes were purified using Moflo XDP cell sorter (Beckman Coulter, USA). 100 ng/mL LPS stimulated monocytes (1 × 10^5^ per well) were incubated at 37 °C for 4 h and were washed with PBS twice to avoid any LPS residue before coculture with neutrophils. OXA-stimulated monocytes (2 × 10^4^ per well) were washed extensively before coculture. Then naïve neutrophils and stimulated monocytes were seeded in a 96-well coculture system with Opti-MEM (Gibco, USA) culture medium for 6 h. The supernatant was collected for the subsequent Gelatin zymography experiment. For siRNA-transfected monocytes, monocytes were cultured in RPMI-1640 medium containing 10% FBS for another 12 h until LPS stimulation, and the following steps were described above.

### Electron transfection of siRNA in monocytes

MACS sorting was used to isolate BM monocytes. Sorted monocytes were transfected with small interference RNA si*Cxcl2* or siNC (final concentration: 1 μM), using the Neon™ Transfection System (Thermo Fisher) at 1950 V, 20 ms, 1 pulse, and then were cultured in RPMI-1640 medium containing 10% FBS for 12 h until LPS stimulation. Four hours after LPS stimulation, the supernatant was collected for analysis of the effect of *Cxcl2* silencing. The Tsingke Chemical Company (Beijing, China) provided the negative control siRNA. And the sequences of siRNA targeting for *Cxcl2* are shown below: si*Cxcl2*-1, CAAAGGCAAGGCTAACTGA; si*Cxcl2*-2, GGGTTGACTTCAAGAACAT; si*Cxcl2*-3, CGCTGTCAATGCCTGAAGA.

### Sample size calculation

The sample size was determined based on the test power, which was used to evaluate the adequacy of the experimental design. A test power of 0.85 or higher was considered acceptable to ensure reliable results. The test power is influenced by several factors, including sample size, effect size, variability, and significance level. In this study, the significance level (α) was set at 0.05. Variability was estimated from the mean square of residuals obtained from the analysis of variance (ANOVA) table. The effect size was defined as the smallest biologically significant difference between the experimental groups, which was determined based on the specific context of the experiment. A smaller sample size generally corresponds to a lower test power; therefore, the sample size was carefully calculated to achieve a test power of at least 0.85.

### Statistics and reproducibility

The statistical analysis in this paper was performed using GraphPad Prism (version 6.01, CA, USA). Significant differences between different groups were determined using two-tailed unpaired Student’s *t*-tests for two-group comparisons and one-way analysis of variance (ANOVA) with Bonferroni’s post hoc test for multiple-group comparisons. Statistical significance was defined as *p* < 0.05 for both the unpaired Student’s *t*-tests and the one-way ANOVA. *P* values (unpaired Student’s *t*-tests) and Bonferroni-corrected *p* values are presented in the figures. The data are shown as means ± SD in Fig. 1, all other data are shown as means ± SEM. In all figures except Fig. 2I, n represents the number of biological replicates; in Fig. 2I, N denotes the total number of cells analyzed using Imaris software. The exact sample size and replication are presented in each figure legend.

### Reporting summary

Further information on research design is available in Nature Portfolio Reporting Summary linked to this article.

## Supplementary information

Supplementary Information

Supplementary Movie

Supplementary Data

Description of Additional Supplementary Files

Reporting summary

## Data Availability

The data supporting the findings of this study are the original experimental record within the article and its Supplementary Information. The uncropped and unedited zymograph images are provided in Supplementary Information, as shown in Supplementary Figs. 10–14. Numerical source data for all graphs and analyzes in the main manuscript can be found in Supplementary Data File. Extra data are available from the corresponding author upon reasonable request.
