# Supplementary material for: The crosstalk of monocyte-neutrophil in hair follicles regulates neutrophil transepidermal migration in contact dermatitis
Source: Commun Biol. 2025 Apr 4;8:564. doi: 10.1038/s42003-025-07960-w (PMC11971313; doi:10.1038/s42003-025-07960-w)
Supplement: Supplementary file 1 — Supplementary Information [file 42003_2025_7960_MOESM1_ESM.pdf]

SUPPLEMENTARY MATERIALS

Title: The crosstalk of monocyte-neutrophil in hair follicles regulates neutrophil transepidermal migration in contact dermatitis

Authors

Zhan Fan,[1] Yilun Xu,[2] Yafang Lu,[3] Xinlin Li,[3] Mengli Xu,[1] Jinxin Liu,[3] Zhenzhen Cai,[2] Jiayang Liu,[2] Jingping Su,[2] Jialu Wang,[3] Qingming Luo,[2]* Zhihong Zhang,[1]* Zheng Liu,[1,2]*

Affiliations

[1]School of Life and Health Sciences, Key Laboratory of One Health of Hainan Province, Collaborative Innovation Center of One Health, Hainan University, Haikou, Hainan 570228, China.

[2]State key laboratory of digital medical engineering, School of Biomedical Engineering, Hainan University, Sanya, Hainan 572025, China

[3]Britton Chance Center and MOE Key Laboratory for Biomedical Photonics, Wuhan National Laboratory for Optoelectronics-Huazhong University of Science and Technology, Wuhan, Hubei 430074, China

*Correspondence: Zheng Liu, liu-zheng@hainanu.edu.cn

Zhihong Zhang, zhzhang@hainanu.edu.cn

Qingming Luo, qluo@hainanu.edu.cn.

Supplementary Figures and Figure Legends

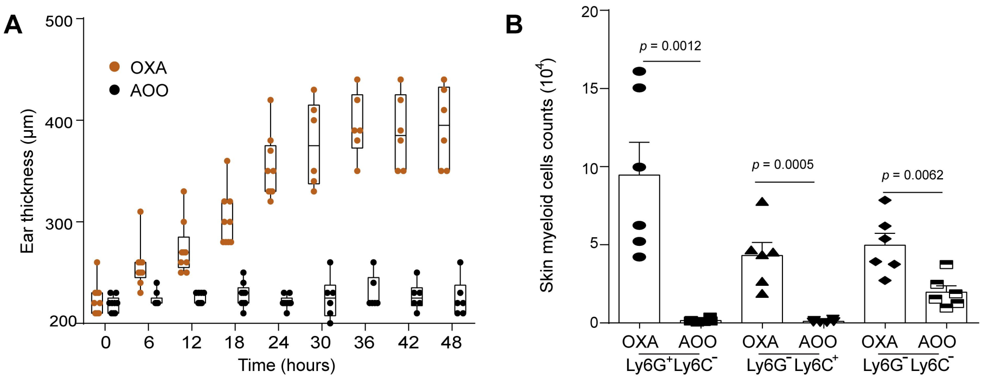

Supplementary Figure 1. OXA-induced inflammation and myeloid cells infiltration in skin. A, Thickness of OXA and vehicle treated ear skin were measured 0-48 hours post OXA or vehicle challenge **B**, Statistical data of skin infiltrated myeloid cells counts 24 hours post OXA or vehicle challenge. $CD45^+CD11b^+Ly6G^+Ly6C^-$ neutrophils and $CD45^+CD11b^+Ly6G^-Ly6C^+$ monocytes counts were compared in OXA and vehicle treated group, n=6, all the mice were female. The error bars indicate SEM and statistical analysis was performed using a two-tailed unpaired Student's *t*-test.

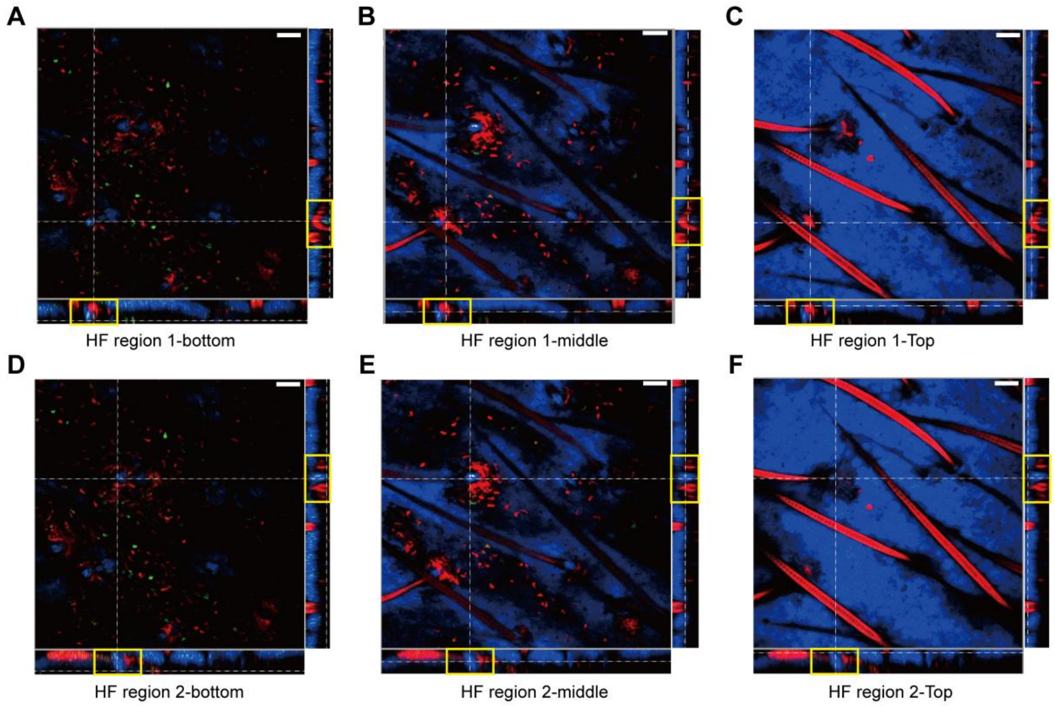

Supplementary Figure 2. **Krt14 expression in hair follicle regions**. Two representative HF regions are displayed vertically from the front and the lateral perspectives, the bottom, middle, and top of HF region 1 and HF region 2 are shown in **A-F**. Scale bar: 50 μm, red: neutrophils, green：monocytes， blue：keratinocytes. Yellow frames indicate the selected HF regions in Z. The data were collected from female mice.

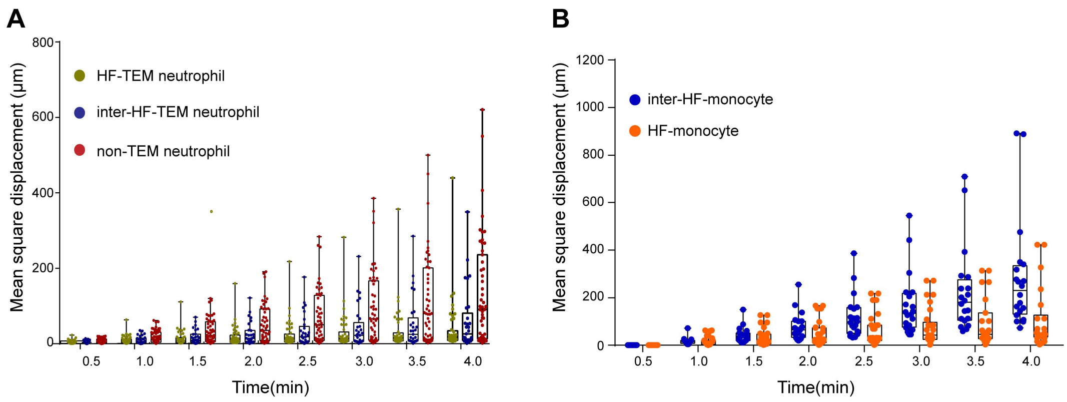

Supplementary Figure 3. The MSD-T of neutrophils and monocytes. A, mean square displacement of HF-TEM neutrophil (n=48), inter-HF-TEM neutrophil (n=29), and non-TEM neutrophil (n=86). **B**, mean square displacement of monocytes in HF (n=29) and inter-HF regions (n=20). The ordinate axis displays the mean square displacement and the abscissa ordinate displays time, the error bars indicate SEM, the data were collected from female mice.

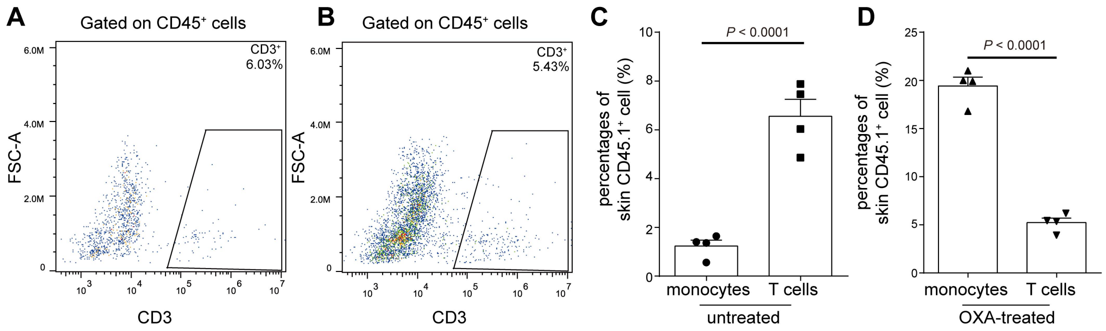

Supplementary Figure 4. T cell and monocytes proportions in untreated and OXA-treated skin . A and B, Gating strategy of $CD45^+CD3^+$ T cell and $CD45^+CD11b^+Ly6G^-Ly6C^+$ monocytes in untreated skin (**A**) or OXA-treated skin (**B**). **C,** the statistical data of $CD45^+CD3^+$ T cells proportions in the untreated skin. **D,** representative flow cytometric plots of $CD45^+CD3^+$ T cell in OXA-treated skin, n=4, all the mice were female. The error bars indicate SEM and statistical analysis was performed using a two-tailed unpaired Student's *t*-test.

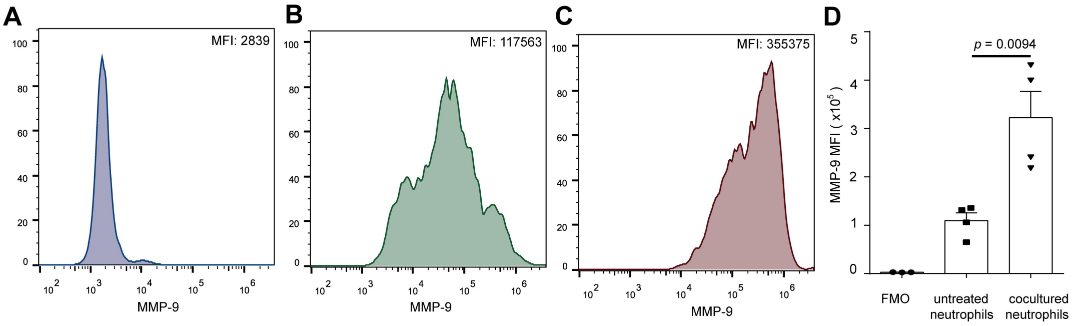

Supplementary Figure 5. The neutrophils in OXA-challenged skin amplify the production of MMP-9 in untreated neutrophils. **A**, the MMP-9 FMO control of CD45.1$^+$ neutrophils. **B** and **C**, the representative histograms displaying the MFI of MMP-9 in untreated CD45.1$^+$ neutrophils (**B**) and cocultured CD45.1$^+$ neutrophils (**C**). **D**, quantitative data of the MMP-9 MFI in **A**, **B,** and **C**; n=4, all the mice were female. The error bars indicate SEM and statistical analysis was performed using a two-tailed unpaired Student's *t*-test.

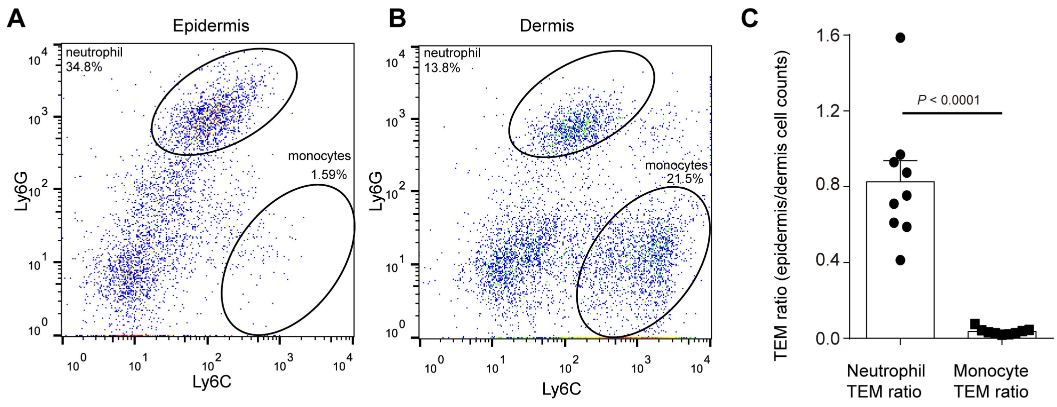

Supplementary Figure 6. The monocyte infiltration in OXA-induced ICD. **A**, the epidermis infiltrated neutrophils and monocytes proportions in OXA-induced ICD. **B**, the dermis infiltrated neutrophils and monocytes proportions in OXA-induced ICD. The flow cytometric plots were gated on $CD11b^+$ cells. **C**, the statistical comparison between neutrophils and monocytes TEM ratio, n=9 , all the mice were female. The error bars indicate SEM and statistical analysis was performed using a two-tailed unpaired Student's *t*-test.

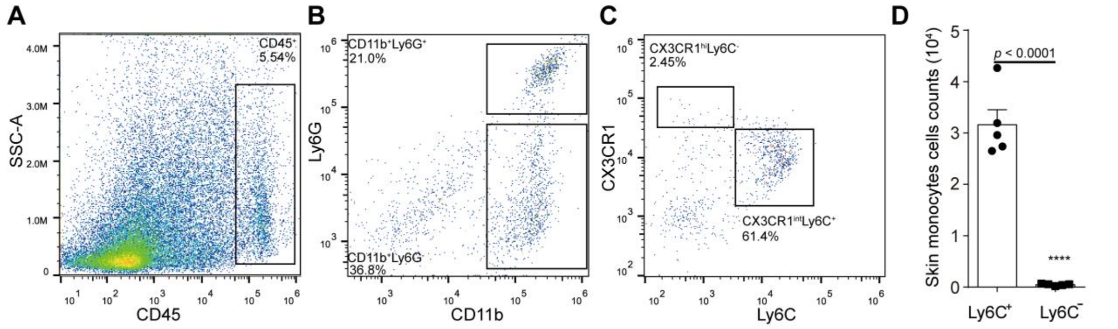

Supplementary Figure 7. The Ly6C$^+$ monocytes and Ly6C$^-$ monocytes counts in OXA-challenged skin. **A**, representative flow cytometric plots of CD45$^+$ cells in OXA-treated skin. **B**, representative flow cytometric plots of CD11b$^+$Ly6G$^+$ and CD11b$^+$Ly6G$^-$ myeloid cells. C, the gating strategy of CD11b$^+$Ly6g$^-$CX3CR1^{hi}Ly6C$^-$ monocytes and CD11b$^+$Ly6G$^-$CX3CR1^{int}Ly6C$^+$ monocytes in skin. **D**, the stastical data of Ly6C$^+$ and Ly6C$^-$ monocytes counts in OXA-challenged skin, n=6, all the mice were female.The error bars indicate SEM and statistical analysis was performed using a two-tailed unpaired Student's *t*-test.

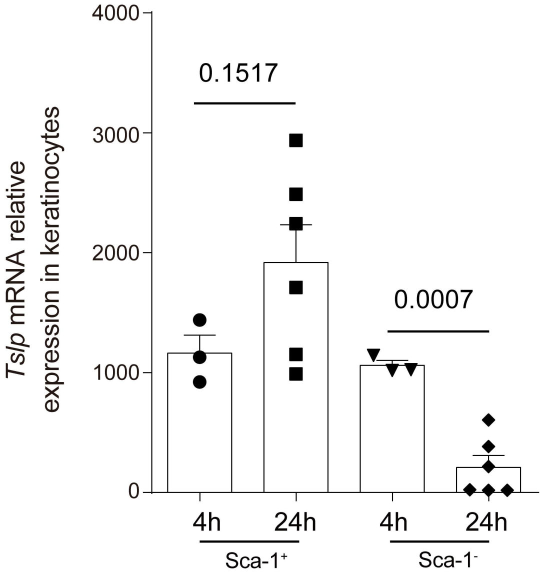

Supplementary Figure 8. The *Tslp* mRNA expression in Sca-1^{+} and Sca-1^{-} keratinocytes 4 hours and 24 hours post OXA challenge. To examine *Tslp* mRNA expression in keratinocytes, qPCR was conducted at 4 hours (n=3) and 24 hours (n=6) post OXA challenge, all the mice were female. Error bars represent the standard error of the mean (SEM), and statistical analysis was performed using a two-tailed unpaired Student’s *t*-test.

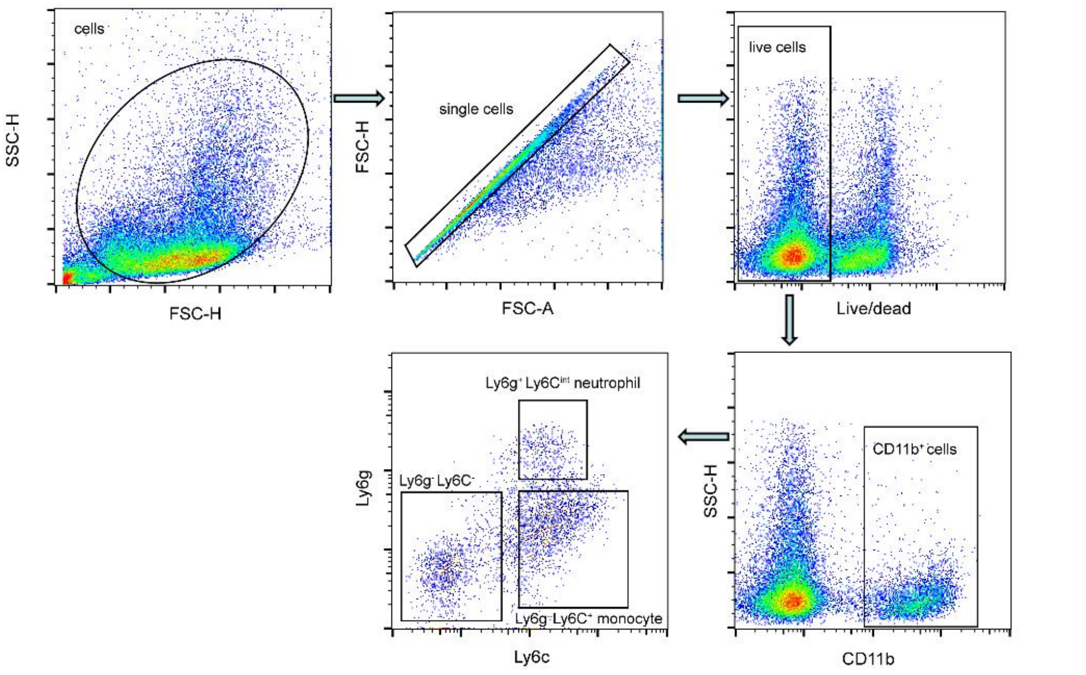

Supplementary Figure 9. The gating strategy for flow cytometry (FACS) plots of skin cells.

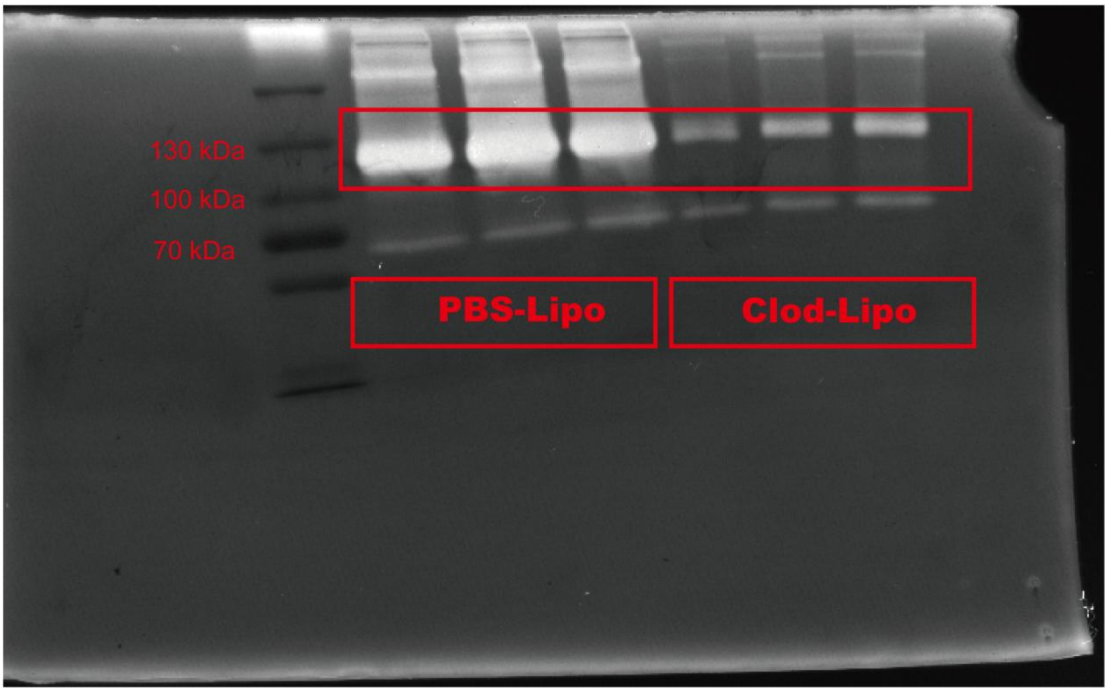

Supplementary Figure 10. Uncropped and unedited zymography image for Figure 4K, 4L.

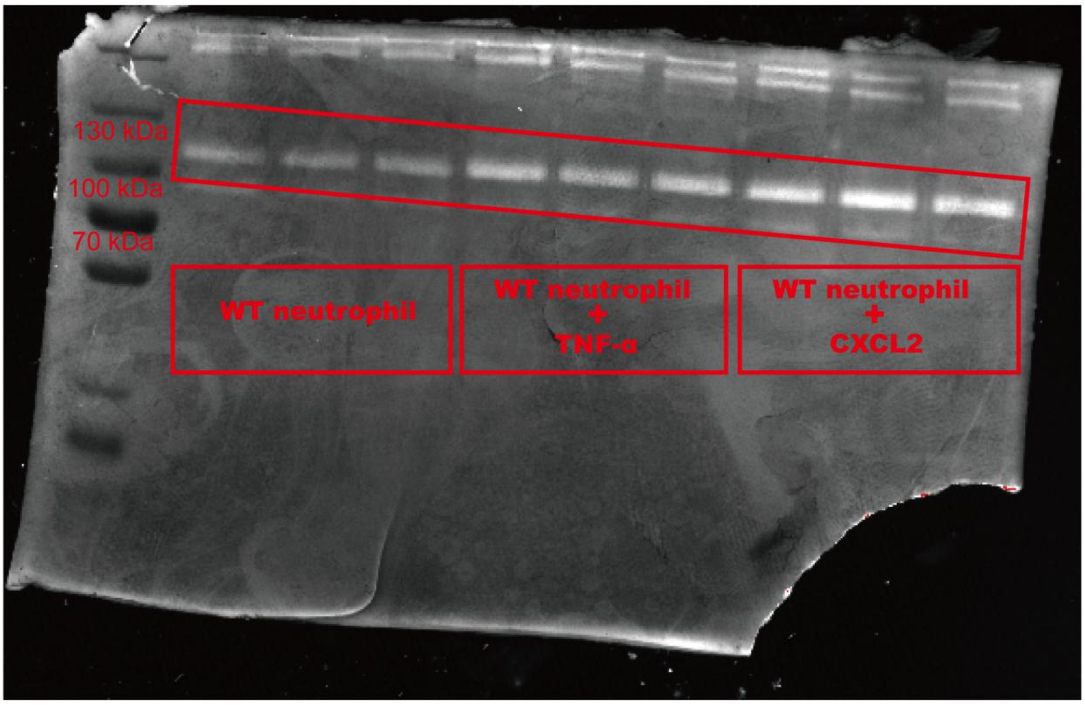

Supplementary Figure 11. Uncropped and unedited zymography image for Figure 6C, 6D.

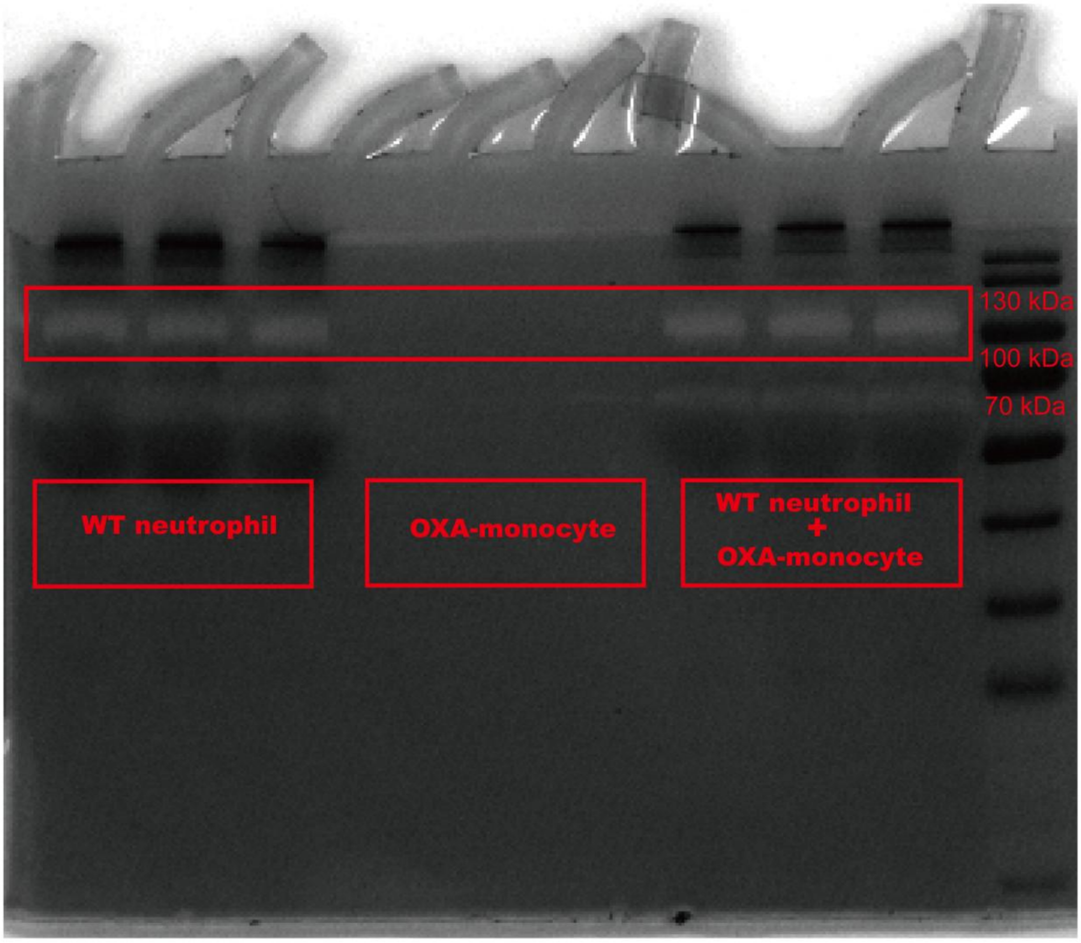

Supplementary Figure 12. Uncropped and unedited zymography image for Figure 6E, 6F.

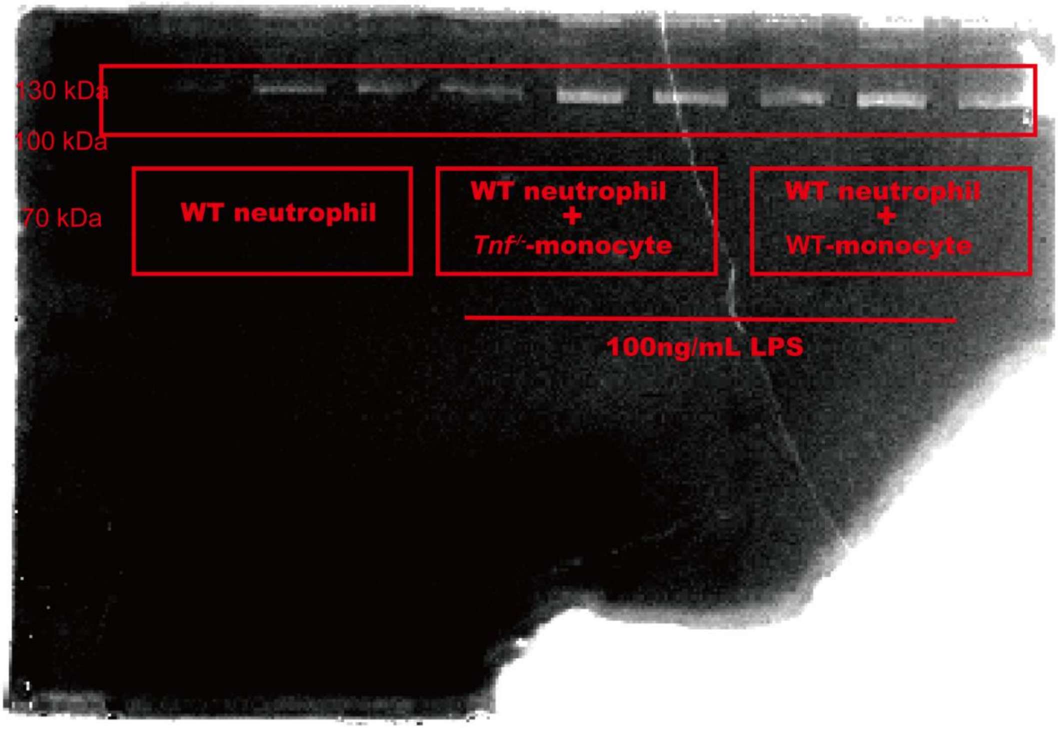

Supplementary Figure 13. Uncropped and unedited zymography image for Figure 6G, 6I.

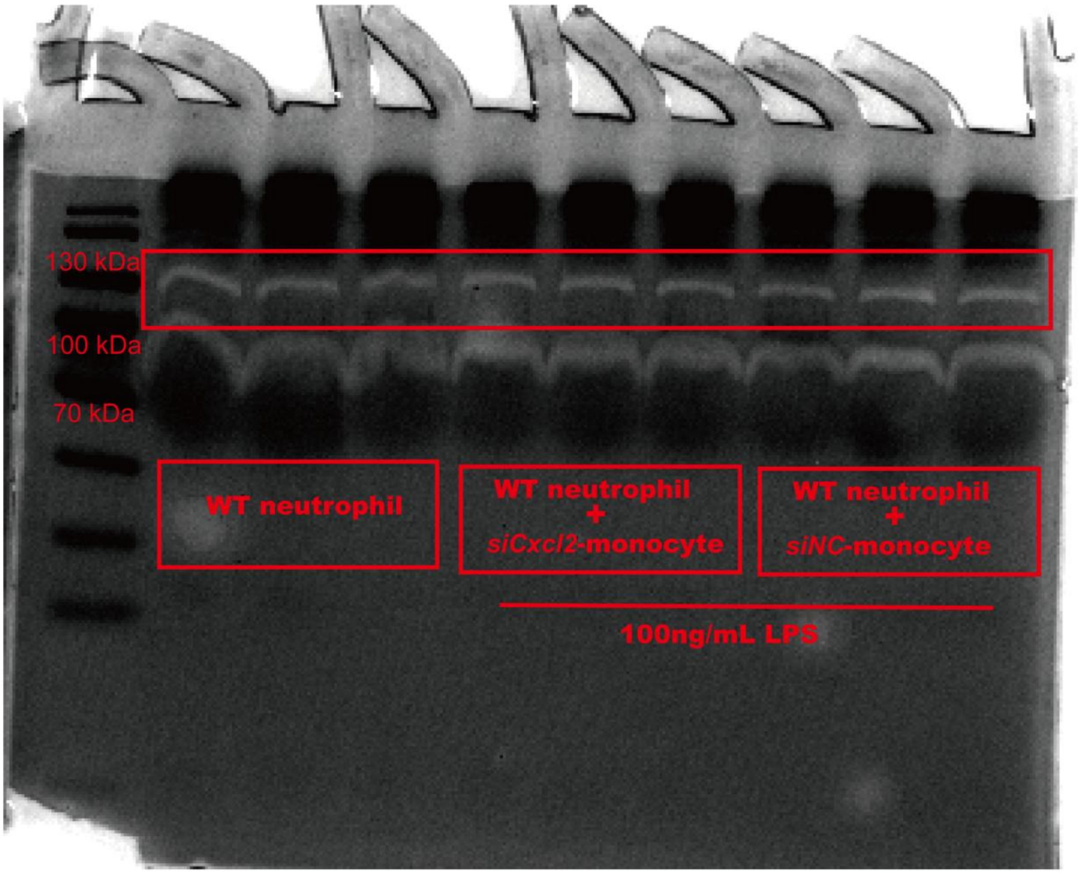

Supplementary Figure 14. Uncropped and unedited zymography image for Figure 6H, 6J.

Reporting on sex and excluded mice:

Animals with major technical complications during experiment were excluded. Specifically, in **Figure.5E**, data from three mice were excluded due to the technical complications. The female mice were used for all the data collection of flow cytometry analyses in irritating contact dermatitis (ICD) models. Female mice were used as recipient of BM chimera and were conducted with ICD. Both female and male mice were used as the donor of BM chimera experiments. Specifically, 6 *Tnf*-deficient male mice were used as BM donors in the BM chimera experiment in **Figure.6A, B**. Female and male mice were used for the data collection of MACS purified neutrophils co-culture experiments, 3 male mice and 3 female mice were used to obtain BM and purify neutrophils *in vitro* in **Figure.6C, D**.
