# Supplementary material for: The crosstalk of monocyte-neutrophil in hair follicles regulates neutrophil transepidermal migration in contact dermatitis
Source: Commun Biol. 2025 Apr 4;8:564. doi: 10.1038/s42003-025-07960-w (PMC11971313; doi:10.1038/s42003-025-07960-w)
Supplement: Supplementary file 4 — Description of Additional Supplementary Files [file 42003_2025_7960_MOESM4_ESM.docx]

Description of Additional Supplementary Files

**File Name:** Supplementary Data

**Description:** The source data behind the graphs in the paper.

**File Name:** Supplementary Movie

**Description:** The intravital imaging displaying RFPlabeled neutrophil migrated toward the HF region within 180 min.
