# Supplementary material for: The crosstalk of monocyte-neutrophil in hair follicles regulates neutrophil transepidermal migration in contact dermatitis
Source: Commun Biol. 2025 Apr 4;8:564. doi: 10.1038/s42003-025-07960-w (PMC11971313; doi:10.1038/s42003-025-07960-w)
Supplement: Supplementary file 5 — Reporting summary [file 42003_2025_7960_MOESM5_ESM.pdf]

nature portfolio

Corresponding author(s): Zhihong Zhang

Last updated by author(s): Mar 18, 2025

Reporting Summary

Nature Portfolio wishes to improve the reproducibility of the work that we publish. This form provides structure for consistency and transparency in reporting. For further information on Nature Portfolio policies, see our Editorial Policies and the Editorial Policy Checklist.

Statistics

For all statistical analyses, confirm that the following items are present in the figure legend, table legend, main text, or Methods section.

n/a	Confirmed	
☐	☒	The exact sample size (*n*) for each experimental group/condition, given as a discrete number and unit of measurement
☐	☒	A statement on whether measurements were taken from distinct samples or whether the same sample was measured repeatedly
☐	☒	The statistical test(s) used AND whether they are one- or two-sided<br>*Only common tests should be described solely by name; describe more complex techniques in the Methods section.*
☒	☐	A description of all covariates tested
☐	☒	A description of any assumptions or corrections, such as tests of normality and adjustment for multiple comparisons
☐	☒	A full description of the statistical parameters including central tendency (e.g. means) or other basic estimates (e.g. regression coefficient) AND variation (e.g. standard deviation) or associated estimates of uncertainty (e.g. confidence intervals)
☐	☒	For null hypothesis testing, the test statistic (e.g. *F*, *t*, *r*) with confidence intervals, effect sizes, degrees of freedom and *P* value noted<br>*Give P values as exact values whenever suitable.*
☒	☐	For Bayesian analysis, information on the choice of priors and Markov chain Monte Carlo settings
☒	☐	For hierarchical and complex designs, identification of the appropriate level for tests and full reporting of outcomes
☒	☐	Estimates of effect sizes (e.g. Cohen's *d*, Pearson's *r*), indicating how they were calculated

Our web collection on statistics for biologists contains articles on many of the points above.

Software and code

Policy information about availability of computer code

Data collection	The Zeiss LSM 780 NLO multiphoto confocal imaging system were used to collect in vivo imaging data; The Bechman Moflo XDP cell sorter was used to sort leukocytes in skin; The Bechman CytoFLEX flow cytometer and the Guava InCyte analyzer were used to perform flow cytometry; The Biorad Gel Doc XR+ documentation system was used to image gelatin zymography .
Data analysis	The CytExpert software vesion 2.3 and FLowJo software V10 documentation were used to collect the flow cytometry data. The Imaris7.6 and Imaris9.0 software were used to trace monocytes, neutrophil and keratinocytes interactions; All statistical analyses were processed by Graphpad Prism software version 6; The image J software (version 2.0.0) was used to analyze gelatin zymography.

For manuscripts utilizing custom algorithms or software that are central to the research but not yet described in published literature, software must be made available to editors and reviewers. We strongly encourage code deposition in a community repository (e.g. GitHub). See the Nature Portfolio guidelines for submitting code & software for further information.

Data

Policy information about availability of data

All manuscripts must include a data availability statement. This statement should provide the following information, where applicable:

- Accession codes, unique identifiers, or web links for publicly available datasets
- A description of any restrictions on data availability
- For clinical datasets or third party data, please ensure that the statement adheres to our policy

The data supporting the findings of this study are the original experimental record within the article and its supplementary information. The uncropped and unedited zymography images are provided in Supplementary information, as shown in Supplementary figure 10-14.Numberical source data for all graphs and analyses in the main manuscript can be found in Supplementary Data File. Extra data are available from the corresponding author upon reasonable request.

Research involving human participants, their data, or biological material

Policy information about studies with human participants or human data. See also policy information about sex, gender (identity/presentation), and sexual orientation and race, ethnicity and racism.

Reporting on sex and gender	No data involving human in this study
Reporting on race, ethnicity, or other socially relevant groupings	No data involving human in this study
Population characteristics	No data involving human in this study
Recruitment	No data involving human in this study
Ethics oversight	No data involving human in this study

Note that full information on the approval of the study protocol must also be provided in the manuscript.

Field-specific reporting

Please select the one below that is the best fit for your research. If you are not sure, read the appropriate sections before making your selection.

☒ Life sciences ☐ Behavioural & social sciences ☐ Ecological, evolutionary & environmental sciences

For a reference copy of the document with all sections, see nature.com/documents/nr-reporting-summary-flat.pdf

Life sciences study design

All studies must disclose on these points even when the disclosure is negative.

Sample size	Sample size was determined to be adequate based on the magnitude and consistency of measurable differences between groups. In addition,the sample sizes of this study were determined on the basis of similar published studies. In trichromatic-fluorescence BM chimera experiments, 9 mice were used to exam the labeling efficiency. In OXA and AOO treated ICD experiments, 6 mice of each group was used. In Clod-liposome or PBS-liposome treated experiments, 12 mice of each group was used. In MMP inhibitor treated and MMP-9 deficient experiments, the sample size for each group was 9 mice. In BM chimera experiments, the sample size for each group was 9. In TNF-α deficient experiments, the sample size for each group was 9 mice. In CXCR2 inhibitor or vehicle-treated experiments, the sample size for each group was 9 mice. In anti-CXCL2 or IgG treated experiments, the sample size for each group was 12 mice. In TNF-α deficient or TNF-α competent BM adoptively transferred chimera experiments, the sample size for each group was 9 mice. In monocyte and neutrophil co-culture and gelatin zymography experiments, 6 wells repeat of each group were used. In CD45.1 and CD45.2 neutrophil co-culture and flow cytometry experiments, 6 OXA-treated CD45.1 mice was used. In the flow cytometry experiment to compare skin infiltrated Ly6C+ and Ly6C- monocytes counts, 6 mice was used. In the flow cytometry experimen to compare skin infiltrated T cell and monocyte, the sample size was 4 mice. In the TSLP mRNA expression qPCR experiment, the sample size was 3 mice.
Data exclusions	Animals with major technical complications during experiments were excluded. Specifically, in Figure. 5F, data from 3 mice were excluded due to the technical complications.
Replication	Every experiment was repeated once or more to ensure every major finding is highly repeatable.
Randomization	To prevent selection bias, mice were randomly allocated to different groups.We selected experiments mice randomly distributed within cages reducing possible biases.
Blinding	The investigators were not blinded to group allocation during data collection. Before the experiment were performed, we have to check the study procedure and analysis methods were correctly done.

Behavioural & social sciences study design

All studies must disclose on these points even when the disclosure is negative.

Study description	*Briefly describe the study type including whether data are quantitative, qualitative, or mixed-methods (e.g. qualitative cross-sectional, quantitative experimental, mixed-methods case study).*
Research sample	*State the research sample (e.g. Harvard university undergraduates, villagers in rural India) and provide relevant demographic information (e.g. age, sex) and indicate whether the sample is representative. Provide a rationale for the study sample chosen. For studies involving existing datasets, please describe the dataset and source.*
Sampling strategy	*Describe the sampling procedure (e.g. random, snowball, stratified, convenience). Describe the statistical methods that were used to predetermine sample size OR if no sample-size calculation was performed, describe how sample sizes were chosen and provide a rationale for why these sample sizes are sufficient. For qualitative data, please indicate whether data saturation was considered, and what criteria were used to decide that no further sampling was needed.*
Data collection	*Provide details about the data collection procedure, including the instruments or devices used to record the data (e.g. pen and paper, computer, eye tracker, video or audio equipment) whether anyone was present besides the participant(s) and the researcher, and whether the researcher was blind to experimental condition and/or the study hypothesis during data collection.*
Timing	*Indicate the start and stop dates of data collection. If there is a gap between collection periods, state the dates for each sample cohort.*
Data exclusions	*If no data were excluded from the analyses, state so OR if data were excluded, provide the exact number of exclusions and the rationale behind them, indicating whether exclusion criteria were pre-established.*
Non-participation	*State how many participants dropped out/declined participation and the reason(s) given OR provide response rate OR state that no participants dropped out/declined participation.*
Randomization	*If participants were not allocated into experimental groups, state so OR describe how participants were allocated to groups, and if allocation was not random, describe how covariates were controlled.*

Ecological, evolutionary & environmental sciences study design

All studies must disclose on these points even when the disclosure is negative.

Study description	*Briefly describe the study. For quantitative data include treatment factors and interactions, design structure (e.g. factorial, nested, hierarchical), nature and number of experimental units and replicates.*
Research sample	*Describe the research sample (e.g. a group of tagged Passer domesticus, all Stenocereus thurberi within Organ Pipe Cactus National Monument), and provide a rationale for the sample choice. When relevant, describe the organism taxa, source, sex, age range and any manipulations. State what population the sample is meant to represent when applicable. For studies involving existing datasets, describe the data and its source.*
Sampling strategy	*Note the sampling procedure. Describe the statistical methods that were used to predetermine sample size OR if no sample-size calculation was performed, describe how sample sizes were chosen and provide a rationale for why these sample sizes are sufficient.*
Data collection	*Describe the data collection procedure, including who recorded the data and how.*
Timing and spatial scale	*Indicate the start and stop dates of data collection, noting the frequency and periodicity of sampling and providing a rationale for these choices. If there is a gap between collection periods, state the dates for each sample cohort. Specify the spatial scale from which the data are taken*
Data exclusions	*If no data were excluded from the analyses, state so OR if data were excluded, describe the exclusions and the rationale behind them, indicating whether exclusion criteria were pre-established.*
Reproducibility	*Describe the measures taken to verify the reproducibility of experimental findings. For each experiment, note whether any attempts to repeat the experiment failed OR state that all attempts to repeat the experiment were successful.*
Randomization	*Describe how samples/organisms/participants were allocated into groups. If allocation was not random, describe how covariates were controlled. If this is not relevant to your study, explain why.*
Blinding	*Describe the extent of blinding used during data acquisition and analysis. If blinding was not possible, describe why OR explain why blinding was not relevant to your study.*

Did the study involve field work? ☐ Yes ☐ No

Field work, collection and transport

Field conditions	*Describe the study conditions for field work, providing relevant parameters (e.g. temperature, rainfall).*
Location	*State the location of the sampling or experiment, providing relevant parameters (e.g. latitude and longitude, elevation, water depth).*
Access & import/export	*Describe the efforts you have made to access habitats and to collect and import/export your samples in a responsible manner and in compliance with local, national and international laws, noting any permits that were obtained (give the name of the issuing authority, the date of issue, and any identifying information).*
Disturbance	*Describe any disturbance caused by the study and how it was minimized.*

Reporting for specific materials, systems and methods

We require information from authors about some types of materials, experimental systems and methods used in many studies. Here, indicate whether each material, system or method listed is relevant to your study. If you are not sure if a list item applies to your research, read the appropriate section before selecting a response.

Materials & experimental systems

n/a	Involved in the study	
☐	☒	Antibodies
☒	☐	Eukaryotic cell lines
☒	☐	Palaeontology and archaeology
☐	☒	Animals and other organisms
☒	☐	Clinical data
☒	☐	Dual use research of concern
☒	☐	Plants

Methods

n/a	Involved in the study	
☒	☐	ChIP-seq
☐	☒	Flow cytometry
☒	☐	MRI-based neuroimaging

Antibodies

Antibodies used

BD Pharmingen:Ly6C (clone: HK1.4,Catalog:569434), Ly6G (clone: 1A8,Catalog:551461), PI(Catalog:550825)
CD11b (clone: M1/70, Catalog:552850), CD45(clone:30-F11, Catalog:557659) .
BioLegend: CD192(clone:SA203G11, Catalog:150605).
eBioscience: Fixable viability dye eFluor780(Catalog: 65-0865-14).
R&D SYSTEMS：Mouse CXCL2/MIP-2 Antibody(Monoclonal Rat IgG2B, Catalog:MAB452-500),
Rat IgG2B Isotype Control(Clone :141945, Catalog: MAB0061)
Proteintech: MMP9 recombinant antibody (Clone: 230069F1, Catalog:82854-5-RR)
Multi-rAb CoraLite@Plus 647-goat anti-Rabbit recombinant secondary antibody(H+L, Cat number:RGAR005)

Validation

Ly6C, Catalog:569434, Isotype:Rat IgG1, κ, Application: FC (Flow Cytometry), The validation has been performed by the manufacturer: https://www.bdbiosciences.com/zh-cn/products/reagents/flow-cytometry-reagents/research-reagents/single-color-antibodies-ruo/alexa-fluor-647-rat-anti-mouse-ly-6c.569434.
Ly6G, Catalog:551461, Isotype: Lewis IgG2a, κ, Application: FC (Flow Cytometry), The validation has been performed by the manufacturer: https://www.bdbiosciences.com/en-us/products/reagents/flow-cytometry-reagents/research-reagents/single-color-antibodies-ruo/pe-rat-anti-mouse-ly-6g.551461.
CD11b, Catalog:552850, Isotype: DA/HA IgG2b, κ, Application: FC (Flow Cytometry), The validation has been performed by the manufacturer:https://www.bdbiosciences.com/zh-cn/products/reagents/flow-cytometry-reagents/research-reagents/single-color-antibodies-ruo/pe-cy-7-rat-anti-cd11b.552850.
CD45, Catalog:557659, Isotype: LOU/M IgG2b, κ, Application: FC (Flow Cytometry), The validation has been performed by the manufacturer:https://www.bdbiosciences.com/zh-cn/products/reagents/flow-cytometry-reagents/research-reagents/single-color-antibodies-ruo/apc-cy-7-rat-anti-mouse-cd45.557659.
PI, Catalog:550825, Application: FC (Flow Cytometry),The validation has been performed by the manufacturer: https://www.bdbiosciences.com/zh-cn/products/reagents/cell-preparation-separation-reagents/blood-lysis/staining-and-cell-preparation/pi-rnase-staining-buffer.550825.
eFluor780, Catalog: 65-0865-14, Application: FC (Flow Cytometry), The validation has been performed by the manufacturer: https://www.thermofisher.cn/order/catalog/product/65-0865-14.
CD192, Catalog:150605,Isotype:Rat IgG2b, κ , Application: FC (Flow Cytometry), The validation has been performed by the manufacturer: https://www.biolegend.com/en-us/explore-new-products/brilliant-violet-421-anti-mouse-cd192-ccr2-antibody-13001?GroupID=BLG14694.
Mouse CXCL2/MIP-2 Antibody, Catalog:MAB452-500, Application:Neutralization, Western Blot, ELISA , The validation has been performed by the manufacturer:https://www.rndsystems.com/cn/products/mouse-cxcl2-grobeta-mip-2-cinc-3-antibody-40605_mab452.
Rat IgG2B Isotype, Catalog:MAB0061, Application:Control, The validation has been performed by the manufacturer: https://www.rndsystems.com/cn/products/rat-igg-2b-isotype-control_mab0061

MMP9 recombinant antibody, Catalog:82854-5-RR, Application:FC(Flow Cytometry), The validation has been performed by the manufacturer: http://www.ptgcn.com/products/Mmp9-Antibody-82854-5-RR.htm
Multi-rAb Coralite@Plus 647-goat anti-Rabit recombant antibody,Catalog:RGAR005,Application:FC(Flow Cytometry),
The validation has been performed by the manufacture: https://www.ptgcn.com/products/Multi-rAb-CoraLite-Plus-647-Goat-Anti-Rabbit-Recombinant-Secondary-Antibody-H-L.htm

Eukaryotic cell lines

Policy information about cell lines and Sex and Gender in Research

Cell line source(s)	*State the source of each cell line used and the sex of all primary cell lines and cells derived from human participants or vertebrate models.*
Authentication	*Describe the authentication procedures for each cell line used OR declare that none of the cell lines used were authenticated.*
Mycoplasma contamination	*Confirm that all cell lines tested negative for mycoplasma contamination OR describe the results of the testing for mycoplasma contamination OR declare that the cell lines were not tested for mycoplasma contamination.*
Commonly misidentified lines (See ICLAC register)	*Name any commonly misidentified cell lines used in the study and provide a rationale for their use.*

Palaeontology and Archaeology

Specimen provenance	*Provide provenance information for specimens and describe permits that were obtained for the work (including the name of the issuing authority, the date of issue, and any identifying information). Permits should encompass collection and, where applicable, export.*
Specimen deposition	*Indicate where the specimens have been deposited to permit free access by other researchers.*
Dating methods	*If new dates are provided, describe how they were obtained (e.g. collection, storage, sample pretreatment and measurement), where they were obtained (i.e. lab name), the calibration program and the protocol for quality assurance OR state that no new dates are provided.*

☐ Tick this box to confirm that the raw and calibrated dates are available in the paper or in Supplementary Information.

Ethics oversight	*Identify the organization(s) that approved or provided guidance on the study protocol, OR state that no ethical approval or guidance was required and explain why not.*

Note that full information on the approval of the study protocol must also be provided in the manuscript.

Animals and other research organisms

Policy information about studies involving animals; ARRIVE guidelines recommended for reporting animal research, and Sex and Gender in Research

Laboratory animals	Wild-type C57BL/6J mice (Female, 8-10 weeks old).<br>B6.129P-Cx3CR1tm1Litt/J, Strain:005582 (Male and female, 6-10 weeks old).<br>B6.129S-Tnftm1Gkl/J ,Strain:005540 (Male and female, 6-10 weeks old).<br>B6.FVB (Cg) -MMP-9tm1Tvu/J , Strain:007084 (Male and female, 6-10 weeks old).<br>B6.129P2-Lyz2tm1(cre)Ifo/J, Strain:004781 (Male and female, 6-10 weeks old).<br>B6.129X1-Gt (ROSA) 26Sortm1(EYFP)Cos/J, Strain:006148 (Male and female, 6-10 weeks old).<br>B6.129(Cg)-Gt (ROSA) 26Sortm4(ACTB-tdTomato,-EGFP)Luo/J, Strain:007676 (Male and female, 6-10 weeks old).<br>Tg (KRT14-cre) 1Amc/J, Strain:004782 (Male and female, 6-10 weeks old).<br>B6.SJL-Ptprca-Pepcb/BoyJ, Strain:002014(female, 10 weeks old).<br>All of the mice were bred and maintained in a specific pathogen-free (SPF) barrier facility at Animal Center of Wuhan National Laboratoryfor Optoelectronics.
Wild animals	The study did not involve wild animals.
Reporting on sex	The female mice were used for the data collection of flow cytometry analyses in irritating contact dermatitis (ICD) models. Female mice were used as recipient of BM chimera and were conducted with ICD. Both female and male mice were used as the donor of BM chimera experiments. Specifically, 6 Tnf-deficient male mice were used as BM donors in the BM chimera experiment in Figure. 6A, B.<br>Female and male mice were used for the data collection of MACS purified neutrophils co-culture experiments, 3 male and 3 female mice were used to obtain BM and purify neutrophils in vitro in Figure.6C, D.
Field-collected samples	The study did not involve samples collected from the field.

Ethics oversight	All animal studies were conducted in compliance with protocols that had been approved by the Institutional Animal Care and Use Committee at Tongji Medical College, Huazhong University of Science and Technology (IACUC number: 844).

Note that full information on the approval of the study protocol must also be provided in the manuscript.

Clinical data

Policy information about clinical studies
All manuscripts should comply with the ICMJE guidelines for publication of clinical research and a completed CONSORT checklist must be included with all submissions.

Clinical trial registration	*Provide the trial registration number from ClinicalTrials.gov or an equivalent agency.*
Study protocol	*Note where the full trial protocol can be accessed OR if not available, explain why.*
Data collection	*Describe the settings and locales of data collection, noting the time periods of recruitment and data collection.*
Outcomes	*Describe how you pre-defined primary and secondary outcome measures and how you assessed these measures.*

Dual use research of concern

Policy information about dual use research of concern

Hazards

Could the accidental, deliberate or reckless misuse of agents or technologies generated in the work, or the application of information presented in the manuscript, pose a threat to:

No	Yes	
☐	☐	Public health
☐	☐	National security
☐	☐	Crops and/or livestock
☐	☐	Ecosystems
☐	☐	Any other significant area

Experiments of concern

Does the work involve any of these experiments of concern:

No	Yes	
☐	☐	Demonstrate how to render a vaccine ineffective
☐	☐	Confer resistance to therapeutically useful antibiotics or antiviral agents
☐	☐	Enhance the virulence of a pathogen or render a nonpathogen virulent
☐	☐	Increase transmissibility of a pathogen
☐	☐	Alter the host range of a pathogen
☐	☐	Enable evasion of diagnostic/detection modalities
☐	☐	Enable the weaponization of a biological agent or toxin
☐	☐	Any other potentially harmful combination of experiments and agents

Plants

Seed stocks	Plants were not involved in this study
Novel plant genotypes	Plants were not involved in this study
Authentication	Plants were not involved in this study

ChIP-seq

Data deposition

☐ Confirm that both raw and final processed data have been deposited in a public database such as GEO.

☐ Confirm that you have deposited or provided access to graph files (e.g. BED files) for the called peaks.

Data access links<br>*May remain private before publication.*	*For "Initial submission" or "Revised version" documents, provide reviewer access links. For your "Final submission" document, provide a link to the deposited data.*
Files in database submission	*Provide a list of all files available in the database submission.*
Genome browser session<br>(e.g. UCSC)	*Provide a link to an anonymized genome browser session for "Initial submission" and "Revised version" documents only, to enable peer review. Write "no longer applicable" for "Final submission" documents.*

Methodology

Replicates	*Describe the experimental replicates, specifying number, type and replicate agreement.*
Sequencing depth	*Describe the sequencing depth for each experiment, providing the total number of reads, uniquely mapped reads, length of reads and whether they were paired- or single-end.*
Antibodies	*Describe the antibodies used for the ChIP-seq experiments; as applicable, provide supplier name, catalog number, clone name, and lot number.*
Peak calling parameters	*Specify the command line program and parameters used for read mapping and peak calling, including the ChIP, control and index files used.*
Data quality	*Describe the methods used to ensure data quality in full detail, including how many peaks are at FDR 5% and above 5-fold enrichment.*
Software	*Describe the software used to collect and analyze the ChIP-seq data. For custom code that has been deposited into a community repository, provide accession details.*

Flow Cytometry

Plots

Confirm that:

☒ The axis labels state the marker and fluorochrome used (e.g. CD4-FITC).

☒ The axis scales are clearly visible. Include numbers along axes only for bottom left plot of group (a 'group' is an analysis of identical markers).

☒ All plots are contour plots with outliers or pseudocolor plots.

☒ A numerical value for number of cells or percentage (with statistics) is provided.

Methodology

Sample preparation	The collected mouse ears were divided into dorsal and ventral parts after the removal of cartilage and incubated in Dispase II (Roche) at 37°C for 1.5 hours. The epidermis and dermis were separated and digested with 0.25 mg mL-1 Liberase TL (Roche) at 37°C for 1.5 hours. The incubated tissue was ground and filtered using a 70 µm nylon mesh to obtain single-cell suspension.
Instrument	CytoFLEX flow cytometer (Beckman Coulter, USA), FACSAria cell sorter Moflo (Beckman Coulter, USA), Guava InCyte analyzer (merck millipore)
Software	The experiment data were collected by CytExpert software version 2.3 (Beckman Coulter, USA) and analyzed by FlowJo software (FlowJo, Ashland,OR, USA).
Cell population abundance	High purity(≥ 90%) of the post-sort fraction (FACS or MACS) was confirmed by flow cytometric analysis.
Gating strategy	Cell clumps are eliminated by FSC-H VS FSC-A gating and live single cells are identified based on the exclusion of a live/Dead dye (PI or eFluor780). Then samples were gated by corresponding marks.

☒ Tick this box to confirm that a figure exemplifying the gating strategy is provided in the Supplementary Information.

Magnetic resonance imaging

Experimental design

Design type	*Indicate task or resting state; event-related or block design.*
Design specifications	*Specify the number of blocks, trials or experimental units per session and/or subject, and specify the length of each trial or block (if trials are blocked) and interval between trials.*
Behavioral performance measures	*State number and/or type of variables recorded (e.g. correct button press, response time) and what statistics were used to establish that the subjects were performing the task as expected (e.g. mean, range, and/or standard deviation across subjects).*

Acquisition

Imaging type(s)	*Specify: functional, structural, diffusion, perfusion.*
Field strength	*Specify in Tesla*
Sequence & imaging parameters	*Specify the pulse sequence type (gradient echo, spin echo, etc.), imaging type (EPI, spiral, etc.), field of view, matrix size, slice thickness, orientation and TE/TR/flip angle.*
Area of acquisition	*State whether a whole brain scan was used OR define the area of acquisition, describing how the region was determined.*

Diffusion MRI ☐ Used ☐ Not used

Preprocessing

Preprocessing software	*Provide detail on software version and revision number and on specific parameters (model/functions, brain extraction, segmentation, smoothing kernel size, etc.).*
Normalization	*If data were normalized/standardized, describe the approach(es): specify linear or non-linear and define image types used for transformation OR indicate that data were not normalized and explain rationale for lack of normalization.*
Normalization template	*Describe the template used for normalization/transformation, specifying subject space or group standardized space (e.g. original Talairach, MNI305, ICBM152) OR indicate that the data were not normalized.*
Noise and artifact removal	*Describe your procedure(s) for artifact and structured noise removal, specifying motion parameters, tissue signals and physiological signals (heart rate, respiration).*
Volume censoring	*Define your software and/or method and criteria for volume censoring, and state the extent of such censoring.*

Statistical modeling & inference

Model type and settings	*Specify type (mass univariate, multivariate, RSA, predictive, etc.) and describe essential details of the model at the first and second levels (e.g. fixed, random or mixed effects; drift or auto-correlation).*
Effect(s) tested	*Define precise effect in terms of the task or stimulus conditions instead of psychological concepts and indicate whether ANOVA or factorial designs were used.*

Specify type of analysis: ☐ Whole brain ☐ ROI-based ☐ Both

Statistic type for inference (See [Eklund et al. 2016])	*Specify voxel-wise or cluster-wise and report all relevant parameters for cluster-wise methods.*
Correction	*Describe the type of correction and how it is obtained for multiple comparisons (e.g. FWE, FDR, permutation or Monte Carlo).*

Models & analysis

n/a	Involved in the study
☐	☐ Functional and/or effective connectivity
☐	☐ Graph analysis
☐	☐ Multivariate modeling or predictive analysis

Functional and/or effective connectivity	*Report the measures of dependence used and the model details (e.g. Pearson correlation, partial correlation, mutual information).*
Graph analysis	*Report the dependent variable and connectivity measure, specifying weighted graph or binarized graph,*

Graph analysis	*subject- or group-level, and the global and/or node summaries used (e.g. clustering coefficient, efficiency, etc.).*
Multivariate modeling and predictive analysis	*Specify independent variables, features extraction and dimension reduction, model, training and evaluation metrics.*
